# Rapid Multi-Well Evaluation of Assorted Materials for Hydrogel-Assisted Giant Unilamellar Vesicle Production: Empowering Bottom-Up Synthetic Biology

**DOI:** 10.3390/gels11010029

**Published:** 2025-01-02

**Authors:** Cherng-Wen Darren Tan, Magdalena Schöller, Eva-Kathrin Ehmoser

**Affiliations:** Institute of Synthetic Bioarchitectures, Department of Bionanosciences, University of Natural Resources and Life Sciences, Vienna, Muthgasse 11, Level 2, 1190 Vienna, Austria; magdalena.schoeller@boku.ac.at (M.S.); eva.ehmoser@boku.ac.at (E.-K.E.)

**Keywords:** giant unilamellar vesicles, hydrogel-assisted, hyaluronic acid, Matrigel, salmon DNA, calcein-AM, artificial cells

## Abstract

Giant unilamellar vesicles (GUVs) are versatile cell models in biomedical and environmental research. Of the various GUV production methods, hydrogel-assisted GUV production is most easily implemented in a typical biological laboratory. To date, agarose, polyvinyl alcohol, cross-linked dextran-PEG, polyacrylamide, and starch hydrogels have been used to produce GUVs. Some leach and contaminate the GUVs, while others require handling toxic material or specialised chemistry, thus limiting their use by novices. Alternative hydrogel materials could address these issues or even offer novel advantages. To facilitate discovery, we replaced the manual spreading of reagents with controlled drop-casting in glass Petri dishes and polystyrene multi-well plates, allowing us to rapidly screen up to 96 GUV-production formulations simultaneously. Exploiting this, we rapidly evaluated assorted biomedical hydrogels, including PEG-DA, cross-linked hyaluronic acid, Matrigel, and cross-linked DNA. All of these alternatives successfully produced GUVs. In the process, we also developed a treatment for recycling agarose and polyvinyl alcohol hydrogels for GUV production, and successfully encapsulated porcine liver esterase (PLE-GUVs). PLE-GUVs offer a novel method of GUV labelling and tracing, which emulates the calcein-AM staining behaviour of cells. Our results highlight the utility of our protocol for potentiating substrate material discovery, as well as protocol and product development.

## 1. Introduction

Giant unilamellar vesicles (GUVs) are synthetic vesicles with typical diameters of 1–300 µm [1,2,3,4]. Their size renders them easily observable using phase contrast microscopy. GUVs are of particular interest because they emulate both the size of cells as well as the supramolecular characteristics of biological membranes, including the mammalian plasma membrane [4,5]. This makes them attractive models for studying cell behaviour or for developing cell-like biosensors and artificial cells in synthetic biology [4,6,7]. GUV membranes comprise a layer of amphiphilic molecules, typically phospholipids or block copolymers [8,9]. Controlled suspension of these amphiphiles in inorganic buffers would cause them to self-assemble into diverse structures, including fluid-filled vesicles bound by a bilayered membrane [1,3,4,10]. When modified with membrane proteins and other functional molecules on their surface, synthetic membranes and vesicles can mimic some of the complex architectures and behaviours of plasma membranes, including receptors, which bind to ligands [11,12,13], and ion channels that regulate the transport of molecules [14,15]. They may even be made to encapsulate enzymes that reconstitute specific metabolisms from cells, such as the phospholipid synthesis pathway [16,17,18,19]. As such, with appropriate modifications, GUVs can serve as surrogates for cells of interest in assays and other processes. This is sometimes necessary, such as when the cells themselves are dangerous to handle, or when the plasma membrane behaviour has to be decoupled from the rest of the cellular physiology, etc. GUVs are also a major area of research in chemical synthetic biology aimed at the development of artificial cells [20,21]. To support these studies, a facile and adequate supply of GUVs is needed. As such, there is considerable interest in developing methods of producing GUVs, especially under physiological conditions.

The three most common methods of producing GUVs are electroformation, microfluidic solvent exchange, and hydrogel-assisted film rehydration [5]. Each approach offers distinct advantages, and each is hindered by unique difficulties. Electroformation uses specialised equipment designed to supply a potential difference between indium tin oxide-coated glass slides [22,23]. When such a glass slide is coated with an organised layer of deposited amphiphiles, the voltage supplied causes the layers to separate and reorganise into GUVs. While this technology has improved considerably over the years, it still requires delicate reagent preparation, and involves complicated handling and operation [24,25]. In addition, the electric fields applied might hinder the encapsulation of useful biomolecules as a consequence of charge separation. On the other hand, microfluidic systems exploit the laminar flow inside microchannels to direct the interaction between aqueous buffers and amphiphiles dissolved in organic solvents [26,27,28]. This method produces GUVs of extremely uniform size. Furthermore, the degree of control over material mixing and interaction also gives users finer control over the composition and modification of the GUVs. However, the microfluidic approach also suffers from the need for specialised equipment. Also, the microchannels often have to be specially designed and cast, since commercially available microfluidic systems tend to have simple geometries inappropriate for GUV formation [28]. This is time consuming and requires specialised skills, including CAD design, photolithography, and soft lithography. Furthermore, the yields from such methods tend to be low, and properly removing residual solvents from the GUVs is challenging [26,27].

A relatively simpler method was proposed by Horger et al. [29] in 2009, which involves the use of a hydrogel to facilitate the formation of GUVs from an ordered lipid deposit [29,30]. Briefly, a layer of low-gelling point agarose hydrogel is quickly dried to form a thin film, and then layers of phospholipids from a chloroform solution are deposited onto its surface. To ensure the even coverage and penetration of the film surface, the solution is spread with a rod or needle until dry. Subsequent addition of a buffer at elevated temperatures then causes rehydration and swelling of the agarose. This facilitates the diffusion of water between the phospholipid layers and pushes them apart. As the outermost layers peel off, they close in upon themselves to form unilamellar vesicles, and later fuse under crowding into GUVs [29]. Unlike electroformation, where the buffer used for GUV formation has to be carefully formulated, Horger’s method allows vesicles to be formed in buffers as diverse as water and physiological salines. This means that GUVs could be produced under conditions and using materials that closely resemble those of cells, thereby allowing them to mimic their biological counterparts more closely. In the following decade and a half, this method underwent minor modifications to suit various needs. Greene et al. adapted this procedure for the production of giant polymer vesicles (polymersomes), produced using the amphiphilic di-block copolymer poly(butadiene)–poly(ethylene oxide) [31]. Nor are we confined to the use of agarose for forming the hydrogel. Only three years before Greene et al. [31], Weinberger et al. [32] showed that polyvinyl alcohol (PVA) can also be used to produce a suitable hydrogel for GUV production [32]. The impetus for this work included the fact that the agarose hydrogels tended to dissolve partially, resulting in the GUV membrane being contaminated with the polysaccharide [32,33]. Unlike agarose, PVA retained its molecular structure under the conditions applied. Also unlike with agarose hydrogels, the lipids did not penetrate the PVA layer, but deposited only on the outer surface. Nonetheless, this approach also produced giant vesicles using diverse buffers. As such, hydrogel-assisted GUV production appears amenable to optimisation. However, we are aware of only a handful of alternatives to agarose and PVA as hydrogel materials for GUV production to date (Table S1). Around the time that Weinberger was investigating PVA, Mora et al. were developing hydrogels produced using cross-linked dextran and poly(ethylene glycol) [34,35]. They reasoned that a hydrogel produced by crosslinking would not be prone to dissolution during the lipid hydration step, unlike agarose and even PVA. More recently, Parigoris et al. reported a similar approach using polyacrylamide hydrogels [36], while Maoyafikuddin et al. returned to the non-cross-linked approach by using starch to produce the substrates [37]. An attractive reason for their use of starch was the desire to use a common and easily available material in biological laboratories. These efforts have helped to increase the range of materials available for GUV production, with each option offering advantages and disadvantages. Researchers only need to decide which combination of materials best suits their experimental demands.

However, most of the alternatives above require handling toxic material, including acrylamide and glutaraldehyde, or specialised chemistry, as is the case for the dextran-poly(ethylene glycol)-based system. These requirements could hinder less experienced researchers who might otherwise be interested in hydrogel research. As such, there is a need to explore other materials for use as substrates. A wider range of available hydrogels would naturally also offer a wider range of advantages, from which users may choose, for meeting specific and even novel challenges.

We started such a search with a consideration of what characteristics allow a substrate to be able to support GUV production. Horger, Weinberger, Mora, and Maoyafikuddin, among others, addressed the possible mechanisms of GUV formation using hydrogels [29,32,34,37]. Apparently, in addition to enhancing hydration of the lipid microenvironment, the swelling of the hydrogels exerts a force normal to the plane of the lipid layer, encouraging swelling and the separation of the lipid bilayers. It stands to reason that any hydrogel capable of imbibing water and swelling would also function, provided that there is no adverse interaction of the lipids with the hydrogel material.

**Figure 1:**
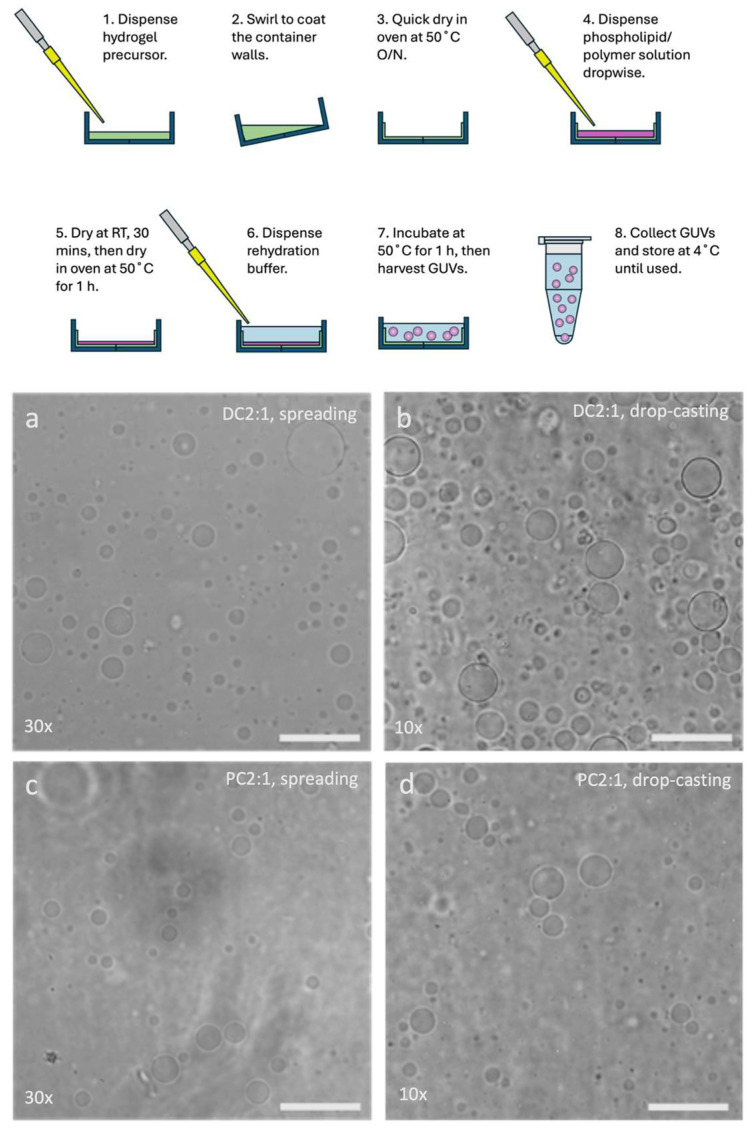
(**Top**) Diagram showing a generic drop-casting approach to hydrogel-assisted GUV production. Step 3 may be modified according to the type of hydrogel or substrate used. O/N: overnight. Note step 2, which creates the protective coat for polystyrene containers, as well as steps 4–5, which modulate the evaporation of chloroform from the amphiphile solution. (**Bottom**) Phase contrast micrographs of DC2:1 (**a**,**b**) and PC2:1 (**c**,**d**) GUVs produced using spreading (**a**,**c**) and drop-casting (**b**,**d**) of AGA hydrogel precursors and amphiphile solutions. Factors (×) indicate how many times the GUV samples have been concentrated. Scale bar = 50 µm.

In order to test this hypothesis, a method of quickly and easily producing diverse hydrogels and GUVs would be necessary. We therefore set ourselves the challenge of developing a platform for performing hydrogel-assisted GUV formation at a higher scale and efficiency. Herein, we report our attempts to facilitate the production and evaluation of different hydrogel and amphiphile formulations, by adapting the hydrogel-assisted GUV formation protocol for use with glass Petri dishes and polystyrene multi-well plates. Our modification replaces reagent spreading with drop-casting of the hydrogel and the coating of solvent-vulnerable vessel walls with a protective hydrogel coat, as well as the measured drop-casting of the amphiphile solution (Figure 1). We show that our approach produces giant unilamellar lipid and polymer vesicles on agarose and PVA hydrogels, of a quality and quantity comparable to and sometimes surpassing that of GUVs produced using a standard reagent-spreading protocol. Facilitated by this multi-well format, we describe screening hydrogels and amorphous gels prepared from a range of materials previously developed for tissue engineering and drug delivery, including cross-linked poly(ethylene glycol) diacrylate (PEG-DA), cross-linked hyaluronic acid (HA), Matrigel, and cross-linked DNA. We show that GUVs can be produced using all of these tested materials. However, the yield of vesicles varied with each substrate, and we observed the incidental entrapment of substrate DNA in GUVs. To further illustrate the ease of handling and relative mildness of our process, we describe encapsulating porcine liver esterase (PLE) in the GUVs. These PLE-functionalised GUVs are able to emulate the calcein-AM staining behaviour of cells, thus offering a novel means of GUV fluorescence analysis. We also show that agarose and PVA hydrogels can be reused at least twice if processed appropriately. We conclude with a discussion of the implications of our findings.

## 2. Results and Discussion

### 2.1. Drop-Cast Agarose and Polyvinyl Alcohol Hydrogels and Drop-Cast Solutions of Lipids and Polymers Can Produce Giant Unilamellar Vesicles (GUVs)

Central to our aim of modifying the hydrogel-assisted GUV production protocol is the incorporation of drop-casting of reagents, such as the hydrogel precursor solution, phospholipid, or polymer solution, in place of spreading them with an implement (Figure 1). To evaluate the efficacy of our process, we compared the GUVs produced from drop-casting with those from conventional protocols, using manual spreading of reagents, as typified by Greene et al. [31]. Where Greene confined GUV production to glass cover slips bounded by a PDMS mould (see Figure S1), we produced the GUVs in glass Petri dishes.

Phase contrast microscopy showed that both methods (spreading vs. drop-casting) using agarose (AGA) as the hydrogel produced GUVs of similar size and lamellarity, from solutions of DOPC and cholesterol (DC2:1) as well as BdEO950 and cholesterol (PC2:1), each in a 2 to 1 molar ratio with respect to cholesterol content (Figure 1). However, visual judgement suggests that the drop-casting method yields a higher number density of GUVs than the spreading approach. This was the case for both types of amphiphile used.

**Figure 2:**
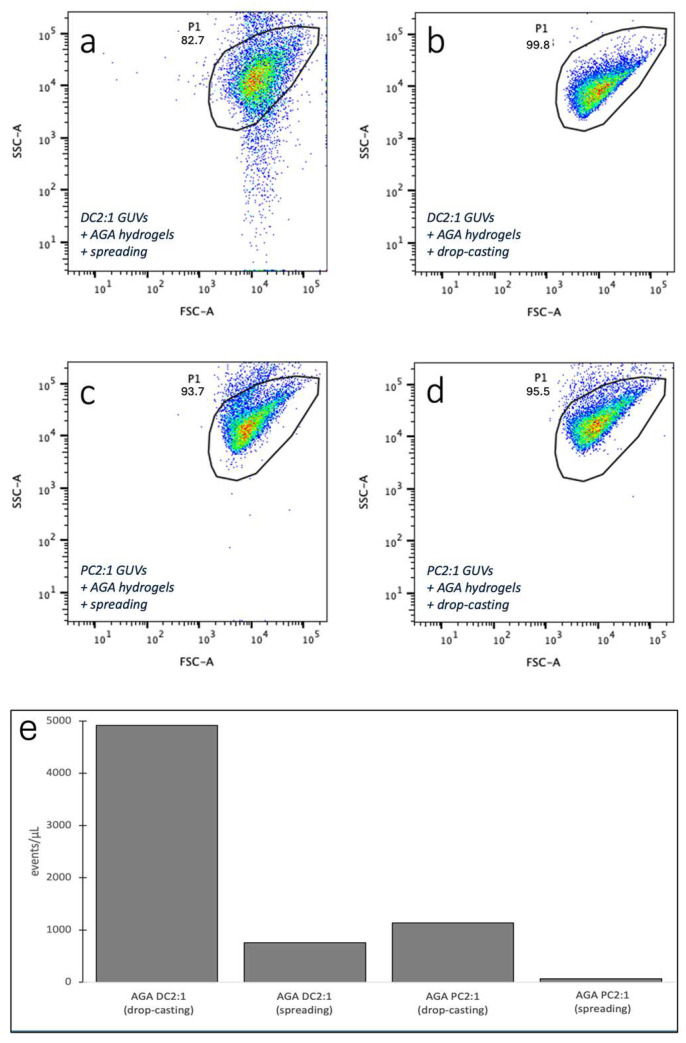
SSC-FSC scatter plots of either DC2:1 (**a**,**b**) or PC2:1 (**c**,**d**) GUVs produced on AGA hydrogels using either spreading (**a**,**c**) or drop-casting (**b**,**d**). Colours indicate regional scatter densities with red being the highest and blue being the lowest. See Methods for how P1 gate was set. (**e**) Bar graphs show yields of GUV (expressed as events/µL as determined by flow cytometry) produced on AGA hydrogels using either DC2:1 or PC2:1, as well as either drop-casting or spreading. The volume of each sample injected was determined by multiplying the flow rate with the time taken to record 10,000 events. The yield or number density of GUVs (events/µL) was then determined by dividing the number of events in the P1 gate by the sample volume injected.

We also used analytical flow cytometry to characterise GUV size and complexity. GUVs produced using both methods presented >80% of events in the P1 gate previously determined to likely represent GUVs of a similar character (Figure 2). The more condensed scatter observed for the GUVs produced using drop-casting, especially for DC2:1, suggests that these GUVs were of a more uniform character than those produced using spreading. In fact, DC2:1 vesicles showed a pronounced polydispersity in side scatter when the spreading method was used. This suggests differences in GUV complexity, perhaps due to differences in lamellarity, although this was difficult to discern in microscopy.

Furthermore, a comparison of number densities also showed a 5-fold and 17-fold increase for DC2:1 and PC2:1 GUVs, respectively, when drop-casting was used (Figure 2).

Taken together, the data suggest that the drop-casting agarose, phospholipid, and di-block copolymer in a glass Petri dish allows for the production of GUVs comparable to and in some cases better than those from the spreading method.

**Figure 3:**
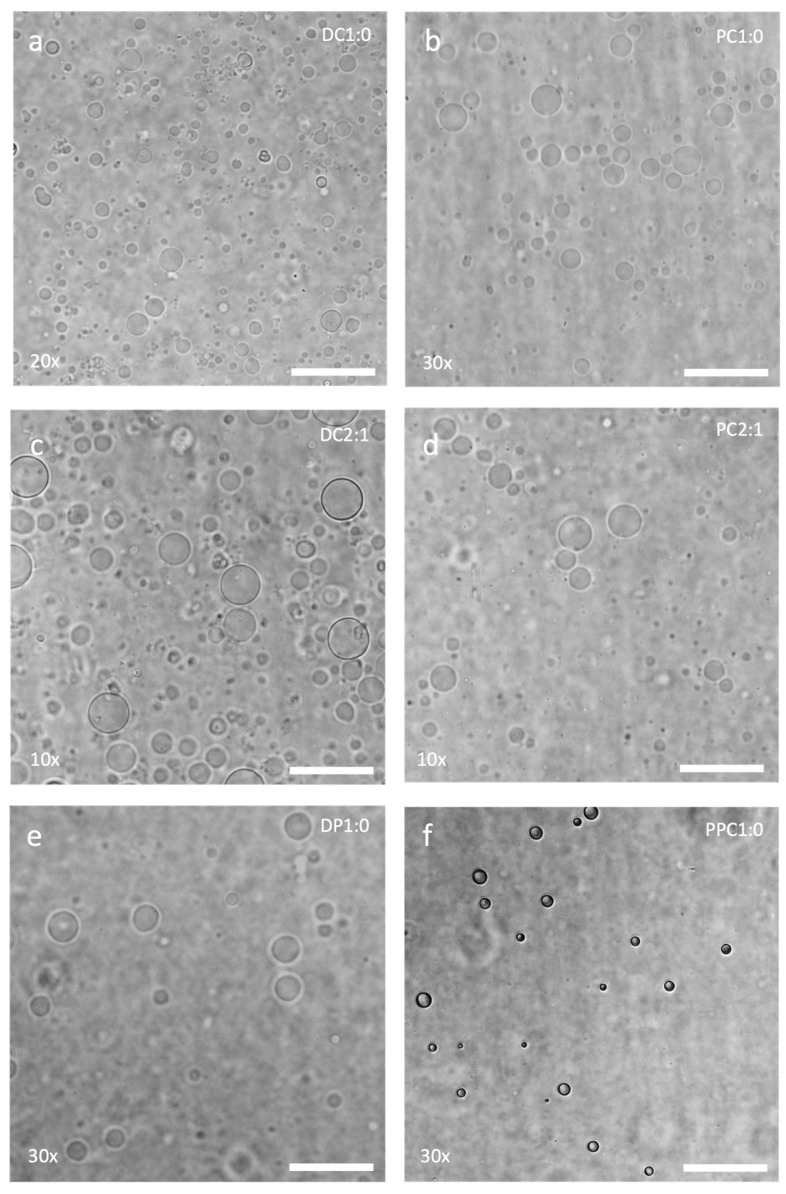
Phase contrast micrographs of GUVs made from (**a**) DC1:0, (**b**) PC1:0, (**c**) DC2:1, (**d**) PC2:1, (**e**) DP1:0, and (**f**) PPC1:0 on AGA. Factors (×) indicate how many times the GUV samples have been concentrated. Scale bar = 50 µm.

We then attempted to produce GUVs from other formulations of DOPC, BdEO950, and cholesterol on AGA hydrogels, as well as hydrogels made from polyvinyl alcohol (PVA) using the drop-casting method in glass Petri dishes. DOPC and cholesterol mixtures with molar ratios of 1:0 (DC1:0) and 2:1 (DC2:1), as well as BdEO950 and cholesterol mixtures with molar ratios of 1:0 (PC1:0) and 2:1 (PC2:1), were compared. We also evaluated solutions of dipalmitoylphosphatidylcholine (DP1:0) and PEG-PLA (PPC1:0). Phase contrast microscopy showed that all amphiphile formulations were able to produce GUVs on AGA hydrogels, although to varying degrees in terms of yield (Figure 3). In general, DC1:0, DC2:1, PC1:0, and PC2:1 appeared to produce more GUVs than DP1:0 and PPC1:0.

**Figure 4:**
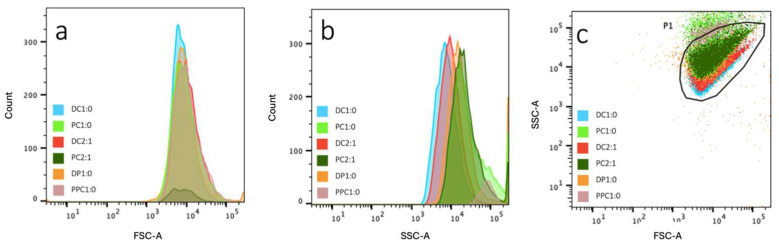
Overlay of (**a**) FSC and (**b**) SSC histograms, as well as (**c**) SSC-FSC scatter plots of GUVs produced from DC1:0, PC1:0, DC2:1, PC2:1, DP1:0, and PPC1:0 on AGA hydrogels.

A comparison of the SSC-FSC scatter plots also showed that the GUVs produced have diverse size and complexity characteristics (Figure S2). While DC1:0, DC2:1, DP1:0, PC1:0, and PC2:1 all presented >80% events in the P1 gate, PPC1:0 presented a scatter plot distinctly different from those of the others. Although the forward scatters suggest that the size distribution is similar between the GUVs, the side scatter comparison suggests variation in complexity between them (Figure 4). The results obtained when PVA hydrogels were used are similar to those from the AGA hydrogels (Figures S3–S5).

### 2.2. Drop-Casting in Glass Petri Dishes and Arraying in a 6-Well Plate Emulates the Multi-Well Format

To demonstrate the ease of handling afforded by the drop-casting method, we drop-cast AGA, PVA, and mixtures of agarose and polyvinyl alcohol (AGA-PVA) into glass Petri dishes set into a 6-well plate, in order to emulate the process were it to be performed entirely in a multi-well plate. We then used the hydrogels produced to drop-cast DC2:1 and PC2:1 for producing GUVs. The same reaction conditions were used for all formulations. As before, hydrogels comprising only AGA and PVA produced GUVs, with DC2:1 generally resulting in a higher yield than PC2:1 (Figure S6). In addition, the hydrogels produced from mixtures of AVA and PVA also produced GUVs comparable to those from the pure hydrogels (Figures S6 and S7). AGA to PVA volume ratios of 2:1 (AGA-PVA 2:1), 1:1 (AGA-PVA 1:1), and 1:2 (AGA-PVA 1:2) were evaluated.

Flow cytometry showed that the GUVs produced from all the hydrogel types presented >80% of events in the P1 gate (Figure S8). In general, there was a higher yield of DC2:1 than PC2:1 GUVs, with the AGA-PVA hydrogels producing higher number densities of DC2:1 GUVs than the other formulations (Figure S9).

A comparison of the forward scatter from the different samples shows that the size distribution is similar between the GUVs prepared from the different hydrogels and membrane materials (Figures S10 and S11). On the other hand, the side scatter comparison suggests an increase in DC2:1 GUV complexity when PVA and AGA-PVA were used as the hydrogel.

**Figure 5:**
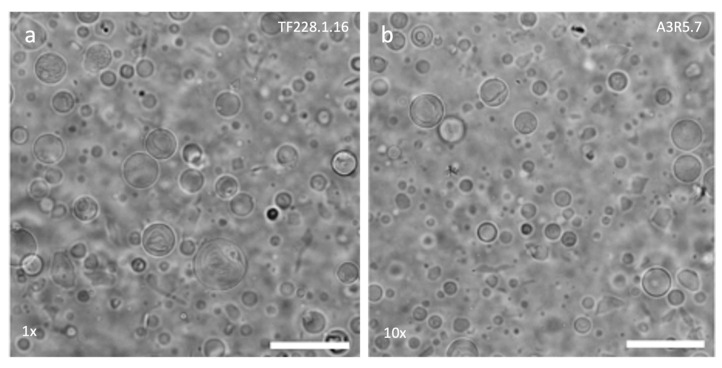
Phase contrast micrographs of GUVs produced on AGA-PVA 1:1 hydrogels using total lipids extracted from (**a**) TF228.1.16 and (**b**) A3R5.7 cells. Micrographs are representative of duplicate experiments. Factors (×) indicate how many times the GUV samples have been concentrated. Scale bar = 50 µm.

Taken together, the data show that a multi-well format would allow for the quick preparation and evaluation of hydrogel-supported GUV samples. This allowed us to also rapidly evaluate amphiphile formulations and rehydration buffers for diverse applications. Since AGA-PVA 1:1 appeared to perform better than the other hydrogel formulations, it was used to evaluate the ability of lipid extracts from mammalian cells to form GUVs, as well as the amenability of our protocol to enzyme encapsulation in the GUVs (Section 2.6). Phase contrast microscopy showed that the total lipids extracted from both A3R5.7 and TF228.1.16 cells were able to produce GUVs on AGA-PVA 1:1 hydrogels (Figure 5). However, a different volume of lipid solution was used since we had not determined the mass or types of lipids present in the extracts, especially of the phospholipids. Nonetheless, the yield of vesicles, especially from TF228.1.16, was comparable to that from some of the copolymers tested.

### 2.3. Polystyrene Multi-Well Plates Can Be Used for Drop-Casting Hydrogel Precursors and Lipid Solutions for GUV Production

**Figure 6:**
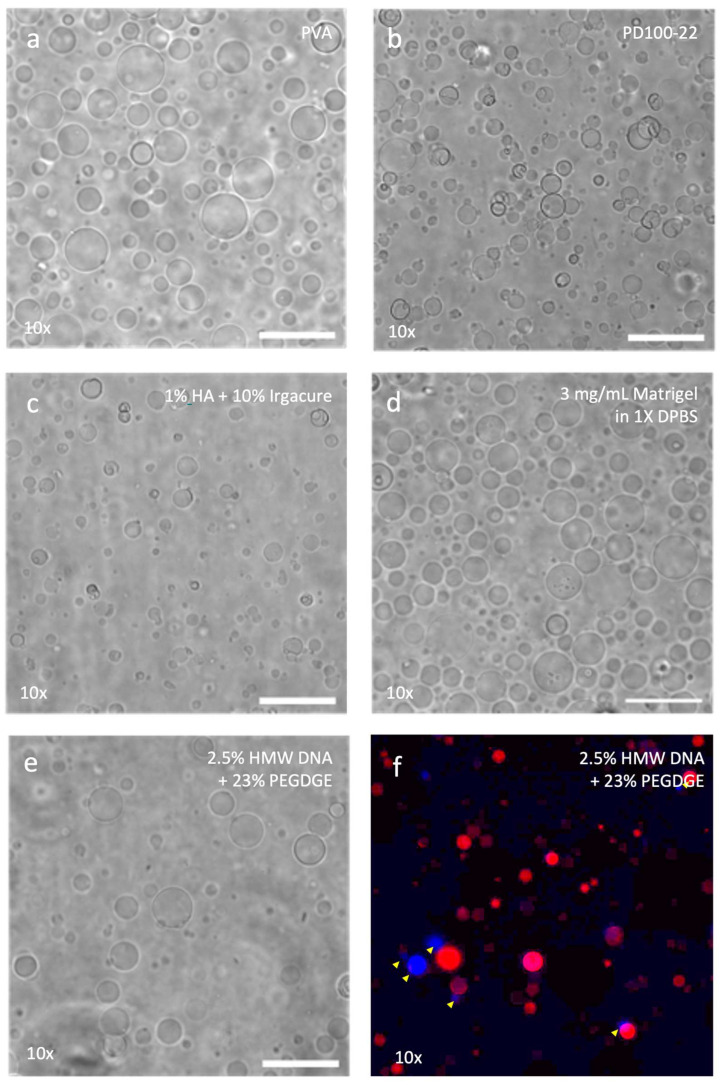
Phase contrast micrographs showing DC2:1 GUVs produced in polystyrene multi-well plates using (**a**) PVA, (**b**) PD100-22, (**c**) 1% HA + 10% Irgacure, (**d**) 3 mg/mL Matrigel, and (**e**) 2.5% HMW DNA + 23% PEGDGE hydrogels. Each figure shown is only a representative of the dataset being reported. Please see the relevant Supplementary Figures for the rest of the micrographs. (**f**) Epi-fluorescence micrograph of DC2:1 + Rh-PE GUVs (red) stained with Hoechst stain (blue). Arrowheads indicate GUVs encapsulating DNA material. Factors (×) indicate how many times the GUV samples have been concentrated. Scale bar = 50 µm.

While the use of glass Petri dishes in a 6-well plate shows that our drop-casting protocol can, in principle, be adapted for a multi-well format, it still suffers from the fact that commercially available glass Petri dishes only fit a 6-well plate. We wanted to obviate the need for glass Petri dishes, as well as to be able to scale down the platform, in order to accommodate the testing of limited or expensive material. A major obstacle to the use of polystyrene is its vulnerability to dissolution by chloroform. As such, modifications to the drop-casting protocol, including the partial coating of the well walls with hydrogel precursors as well as the dropwise application of the lipid and polymer solutions (described in Methods), had to be made. When the modified procedure was carried out in a 24-well plate, we found that AGA, PVA, and AGA-PVA were still able to produce the desired hydrogels. Furthermore, DC2:1 and PC2:1 both produced GUVs in the wells, with PC2:1 again appearing to yield lower number densities (Figure 6a and Figure S12). More importantly, no visible debris similar to that produced in chloroform-treated polystyrene was observed. This demonstrated the feasibility of using the drop-casting approach for GUV production in polystyrene multi-well plates, provided that care was taken to partially coat the walls. We therefore used both 24-well and 96-well plates for subsequent GUV production.

### 2.4. Cross-Linked PEG-DA, Hyaluronic Acid, Matrigel, and DNA Hydrogels Can Be Used for GUV Production

PEG-DA, hyaluronic acid, Matrigel, and DNA hydrogels were evaluated for their ability to form DC2:1 GUVs in multi-well plates. The formulations of PEG-DA tested include 60% PEG-DA cross-linked with 0.15% or 0.37% (*w*/*w*) DMAP (PD60-15 or PD60-37, respectively), 80% PEG-DA cross-linked with 0.11% or 0.28% (*w*/*w*) DMAP (PD80-11 or PD80-28, respectively), and 100% PEG-DA cross-linked with 0.09% (*w*/*w*) or 0.22% (*w*/*w*) DMAP (PD100-09 or PD100-22, respectively). All PEG-DA/DMAP formulations used formed hydrogels and GUVs when subsequently coated with DC2:1 and rehydrated (Figure 6b and Figure S13). The highest yield was obtained when using 100% PEG-DA cross-linked with 0.22% DMAP. Preliminary data showed that using increased amounts of DMAP did not increase the GUV yield. As such, hydrogels produced using higher amounts of DMAP were not investigated further.

To prepare hyaluronic acid (HA) hydrogels for GUV production, 1% (methacrylated) HA was reacted with 10%, 30%, and 100% (*w*/*w*) Irgacure in 24- and 96-well plates. After exposure to irradiation at 365 nm, none of the HA formulations had formed a visible hydrogel. After drying, however, they all formed a clear layer at the bottom of the multi-well plates. When these were coated with DC2:1 and subsequently rehydrated, small flakes of soft hydrogel were present for 1% HA reacted with 10% Irgacure, while a mechanically fragile but largely intact hydrogel was left by 1% HA reacted with 30% Irgacure. The most preserved hydrogel left after GUV production was that from 1% HA reacted with 100% Irgacure. Ironically, although all formulations had produced DC2:1 GUVs, it was 1% HA reacted with 10% Irgacure (which had the severely fragmented hydrogel) that presented the highest yield of GUVs (Figure 6c and Figure S14).

When Matrigel was diluted with ultrapure water instead of 1× DPBS (per conventional use), the hydrogels produced tended to be clumpy instead of monolithic. Subsequent coating with DC2:1 supplemented with 0.1 mol% Rh-PE relative to DOPC (DC2:1 Rh-PE) and rehydration with 0.6 Osm/kg sucrose resulted in the partial dissolution of all formulations of Matrigel hydrogels. However, every formulation was also able to produce GUVs, although the quantities and qualities varied (Figure 6d and Figure S15). In general, those hydrogels produced using Matrigel diluted with ultrapure water tended to yield fewer and smaller vesicles. In contrast, 3 mg/mL and 4 mg/mL Matrigel in 1× PBS presented the highest yields of GUVs among the diluted formulations, while undiluted Matrigel presented the highest yields of GUVs among all the materials tested in this work, with vesicles of up to 50 µm in diameter.

DNA hydrogels and amorphous gels were prepared from low-molecular-weight salmon DNA (LMW-DNA) and high-molecular-weight salmon DNA (HMW-DNA), in either single- or double-stranded form. Single- or double-stranded 10% (*w*/*v*) LMW-DNA (ssDNA and dsDNA, respectively) were treated with 5% or 10% (*w*/*w*) PEGDGE and used to prepare substrates. In these cases, reaction with the PEGDGE did not result in a sturdy hydrogel, but in a very viscous, opaque brown, shower gel-like material (amorphous gels). Similar observations were made with single-stranded 10% LMW-DNA when 0.1% or 1% (*w*/*w*) Irgacure was used as the crosslinking photoinitiator instead. Unexpectedly, the drying and coating of these amorphous gels with DC2:1 followed by rehydration also produced DC2:1 GUVs, although they were deformed and appeared to be multilamellar (Figure S16).

When high-molecular-weight salmon DNA was used, 1% and 2.5% HMW-DNA was reacted with 11.5%, 23%, and 34.5% (*w*/*w*) PEGDGE to produce vesicles. These formulations appeared to form a firm hydrogel in 96-well plates, which were subsequently dried, coated with DC2:1, and rehydrated to produce GUVs. Of the six formulations, only those with 23% (*w*/*w*) PEGDGE resulted in appreciable yields of DC2:1 GUVs, while those with 11.5% and 34.5% (*w*/*w*) PEGDGE formed significantly fewer GUVs (Figure 6e and Figure S17).

### 2.5. GUVs Produced on DNA Substrates Can Encapsulate Leached DNA

When this process was repeated with solutions of DC2:1 supplemented with Rh-PE, fluorescent DC2:1 Rh-PE GUVs were produced. As much as possible of the extra GUV buffer was replaced with fresh buffer, before the GUVs were stained with Hoechst 33342 to ascertain the presence and location of contaminating DNA from the substrates. When single- or double-stranded LMW-DNA was used, Hoechst staining was invariably observed both outside and inside the GUVs (Figure S18). This is likely due to the fact that the highly viscous LMW-DNA substrates had almost completely dissolved during rehydration, making its complete removal from the extra-GUV medium difficult. Probing for the presence of Rh-PE in these samples also showed fluorescence in the residual DNA material, suggesting that the phospholipids had likely diffused into the soft substrate. This is reminiscent of the penetration of phospholipids into the dried agarose matrix, as reported by Horger et al. [29].

On the other hand, when HMW-DNA was used for hydrogel preparation, Hoechst staining (blue) was exclusively localised inside the GUVs, suggesting that the vesicles had encapsulated DNA material from the hydrogels (Figure 6f and Figure S19).

**Figure 7:**
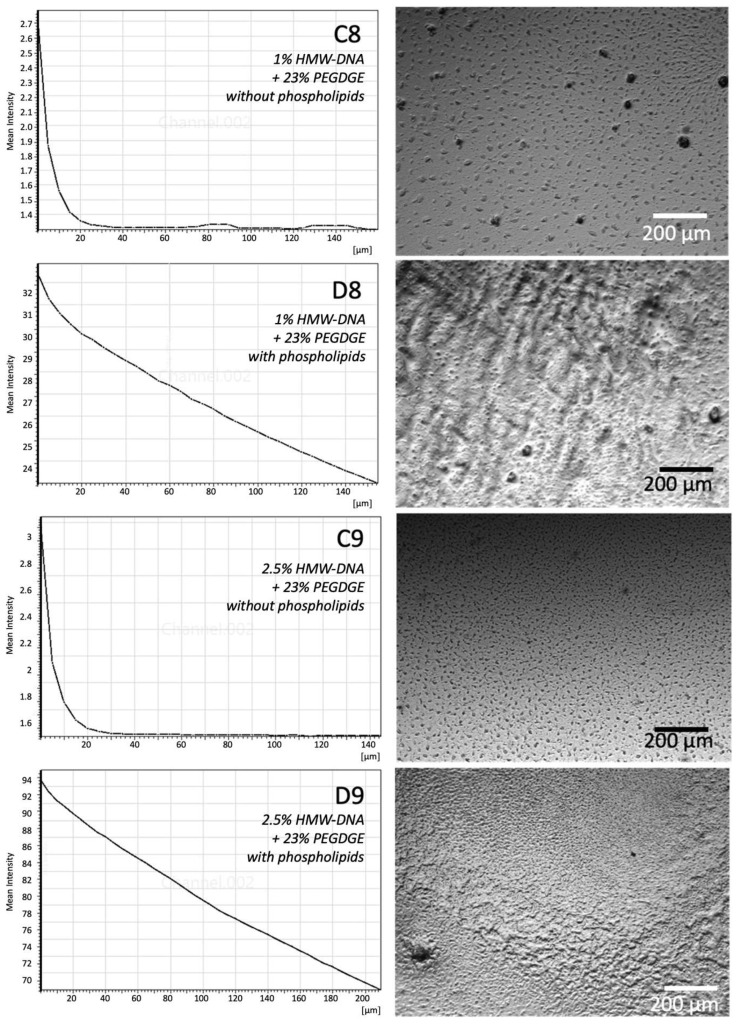
(**Left** column) Z-stacked fluorescence intensity profiles of HMW-DNA and PEGDA hydrogels without (C8, C9) and with (D8, D9) phospholipids. (**Right** column) Corresponding bright field micrograph of the sample surface. Bar = 200 µm. Data shown are representative of triplicates.

Further investigation using z-stacked epi-fluorescence microscopy of the fluorescent phospholipid-coated HMW-DNA hydrogels also showed penetration of Rh-PE into the gel material (Figure 7). Each z-stack was initiated at a plane 50–60 µm above the surface of the gel and terminated at a plane 50–60 µm within the gel. The nearly linear regression of the fluorescence intensity suggests decreasing amounts of Rh-PE with depth, which would happen if the fluorophore had time to diffuse unimpeded into the body of the hydrogel. A distinct difference in surface characteristics was observed between coated and uncoated DNA hydrogels. While a pristine surface appeared smooth with small irregularly spaced surface aggregates, the coated surface presented a rough and irregular surface, where the surface aggregates were largely hidden. These features also differed in size and regularity between the hydrogel formulations used.

### 2.6. GUVs Encapsulating Porcine Liver Esterase Can Emulate Calcein-AM Labelling in Cells

**Figure 8:**
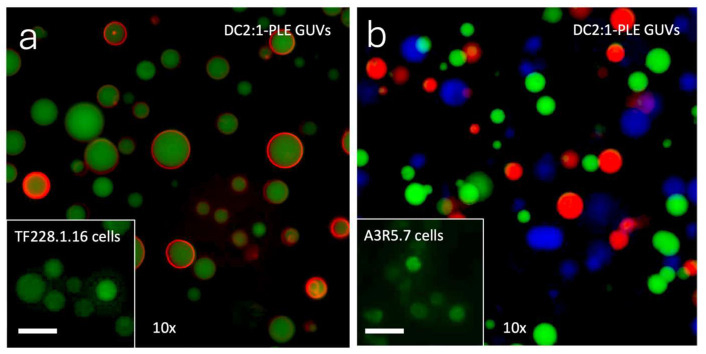
(**a**) Epi-fluorescence micrograph showing DC2:1-PLE GUVs produced using rehydration buffer containing porcine liver esterase (PLE) and labelled with calcein-AM (green) as well as Nile Red (red). Nile Red was used to stain the DC2:1-PLE GUV membrane. Inset: TF228.1.16 cells labelled with calcein-AM. (**b**) Epi-fluorescence image overlay of DC2:1-PLE vesicles labelled with calcein (green), calcein Red (red), and calcein Blue (blue). Inset: A3R5.7 cells labelled with calcein-AM. Factors (×) indicate how many times the GUV samples have been concentrated. Scale bar = 50 µm.

AVA-PVA 1:1 was also used to produce DC2:1 GUVs encapsulating porcine liver esterase (PLE) (DC2:1-PLE). Phase contrast microscopy showed that the presence of PLE in the rehydration buffer did not hinder the formation of GUVs, although the yield appeared to be lower than that of DC2:1 GUVs produced in the absence of PLE. The labelling of DC2:1-PLE with calcein-AM resulted in the intra-GUV fluorescence of trapped calcein, mimicking that observed by cells stained with the same probe (Figure 8a, insets). Indeed, DC2:1-PLE GUVs could also be labelled by calcein-AM Red and calcein-AM Blue. When mixed, DC2:1-PLE GUVs labelled by all three probes separately present three GUV populations of distinctly different fluorescence profiles (Figure 8b). The results demonstrate that the PLE retained its ability to cleave acetoxymethylester from calcein-AM, despite having been subjected to the conditions of GUV formation.

### 2.7. Agarose, Polyvinyl Alcohol, and Agarose–Polyvinyl Alcohol Combination Hydrogels Can Be Reused Effectively to Produce GUVs

**Figure 9:**
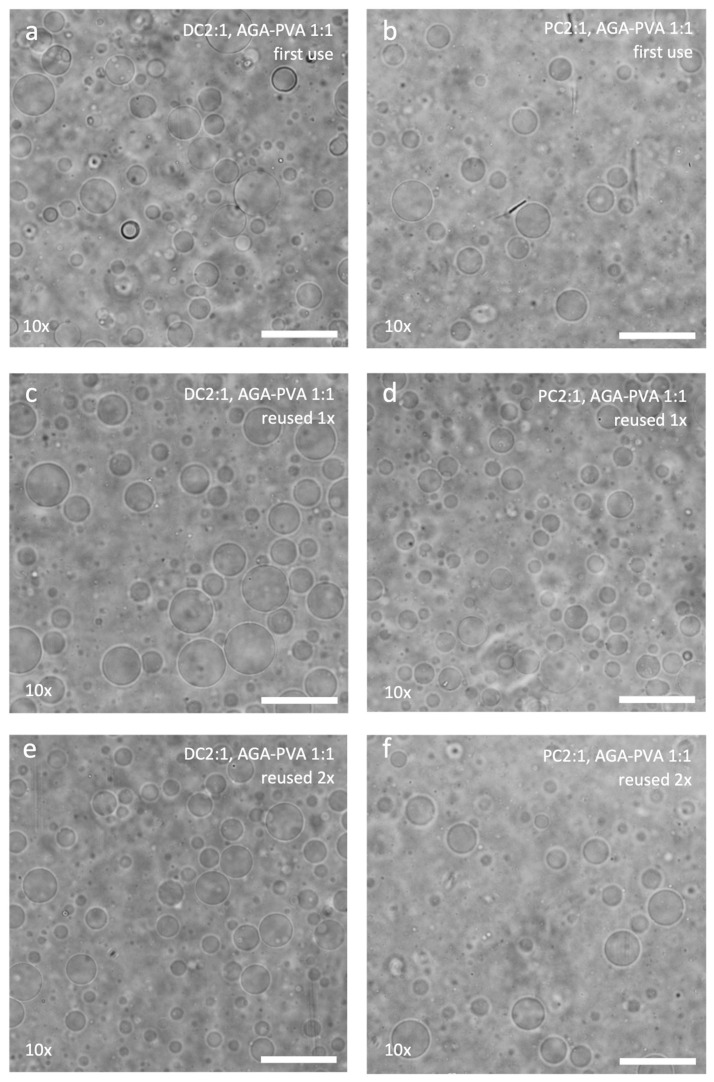
Phase contrast micrographs showing GUVs produced from DC2:1 (**a**,**c**,**e**) and PC2:1 (**b**,**d**,**f**) on AGA-PVA 1:1, used (**a**,**b**) for the first time, (**c**,**d**) reused once, and (**e**,**f**) reused twice. Factors (×) indicate how many times the GUV samples have been concentrated. Scale bar = 50 µm.

Finally, the multi-well format allowed us to conveniently handle the hydrogels during mass exposure to varying conditions. In this way, we were able to subject used hydrogels of different types to washing and drying cycles designed to extract excess phospholipid as well as sucrose from them. These were then used again for drop-casting DC2:1 and PC2:1, followed by thin film rehydration. Hydrogels of AGA, PVA, and AGA-PVA 1:1 were all able to produce GUVs even after having been subjected to two rounds (batches) of reuse (Figure 9).

Analytical flow cytometry showed that except for PC 2:1 produced on AGA:PVA 1:1, the yield of GUVs tended to decrease when the hydrogels were reused (Figure S20). However, the SSC-FSC scatter plot showed >80% of events in the P1 gate for all samples, indicating the similarity of their size and complexity (Figure S21).

When producing giant unilamellar vesicles, it is desirable for the protocol to produce vesicles of minimal size variation, comprising only a single bilayered membrane (unilamellarity), and of high yield [5,22]. This is necessary to ensure the predictable bulk behaviour of the vesicles, and that sufficient quantities are available for useful work. However, these were not our aims, and they lie outside the scope of this work.

What we were primarily interested in was whether assorted hydrogels developed for other uses would also be able to support thin film rehydration for GUV production. This would show that there is potential for expanding the repertoire of substrates available to GUV researchers, thus allowing them to cater to specific challenges.

However, since the selected materials were developed for other purposes, we had to ascertain which formulations of each were appropriate for our needs. In order to facilitate the evaluation of multiple materials, each presenting multiple reaction formulations, we thought to adapt the conventional protocol for hydrogel-assisted GUV production, in order for it to have shorter or simpler steps and easier handling, as well as multiplicity. We achieved those advantages by predominantly using drop-casting as the method for applying thin film precursors, including those for the eventual hydrogel as well as amphiphile layers, and by adapting the protocol to a multi-well format. Although Mora et al. [34] reported using drop-casting as well, it is not common when compared to manual spreading of the film solutions. Spreading with a needle or a pipette is generally preferred when applying the lipid or polymer solution to the hydrogel surface, in order to avoid uneven deposition of the amphiphiles. In contrast, as the solvent (typically chloroform) evaporates without manual spreading, the initially uniform solution layer tends to shrink into droplets or finger-like projections, in what is called the Marangoni effect [38]. This is thought to result in uneven [25] or disordered deposition of the amphiphiles, thus impeding hydration of the hydrophilic domains and the organisation of the molecules into appropriate membrane bilayers or monolayers (as in the case of tri-block copolymers [13,39]). We show that this impediment might be alleviated by controlling the solvent evaporation, such as we have done by first allowing the solutions to evaporate slowly at room temperature, before subjecting the deposits to elevated temperatures and air circulation in order to remove residual solvent. In fact, our data suggest that drop-casting results in greater yields and comparable quality of GUVs when compared to the conventional spreading approach.

We chose DOPC and DPPC as phospholipids of interest given their prevalence in mammalian cell membranes. In addition, most of the formulations used included cholesterol in a mole fraction, which models that typically found in mammals. We used these mixtures for GUV production to increase the cell-like character of our vesicles. In contrast, we picked two synthetic copolymers to reiterate that hydrogel-assisted GUV production is also amenable to polymersome production. Our group is familiar with the use of poly(ethylene oxide)–poly(butadiene) (BdEO1800 of average molecular weight 1800 g/mol) for the fabrication of nano-sized polymersomes [11,40,41]. This polymer is also of the same type (although of lower molecular weight) as that investigated by Greene et al. [31] for hydrogel-assisted GUV production. Here, we use the variant BdEO that is half the average molecular weight of BdEO1800. We surmise that a BdEO950 (average molecular weight of 950 g/mol) bilayered membrane would have a thickness closer to that reported for a phospholipid bilayer. We argue that among BdEO polymers a BdEO950 polymersome would emulate the physical characteristics of a cell membrane more accurately, hence its selection. PEG-PLA, on the other hand, is one of a group of di-block copolymers actively researched as a biomedical implant material [42,43]. We included it to show that even such well-established materials might find additional value in GUV research.

While not always effective (in terms of yield and generating minimal debris) when certain di-block copolymers were used, we have shown that drop-casting of lipid solutions did not result in an appreciable increase in debris, nor in a loss of GUV quality and quantity. In fact, we observed lower yields of GUV when using the spreading method. This is probably due to there being a smaller surface area of phospholipids available for rehydration, as well as possible adsorption of amphiphile material to the spreading tools, resulting in the loss of membrane-forming mass. Drop-casting could have circumvented these problems. We point out that we were able to produce vesicles even from the typically recalcitrant PEG-PLA [44]. We defined the relative quality of vesicles in terms of the forward (FSC) and side scatter (SSC) presented by analytical flow cytometry. FSC served as an indicator for relative size, while SSC indicated GUV complexity, such as lamellarity and surface texture [45,46]. We note from previous reports that the use of forward scatter, at least for analysis of size, is not reliable, and that the Coulter Principle, which relies on particle charge density, is preferred [34,36]. We also note that since the BD FACSCanto II™ uses differential pressure sample injection, our measurements of sample yield would not be as accurate as those from a system employing volumetric injection [47]. In our case, however, we did not aim for precise quantitative comparisons between samples. Flow cytometry, supplemented with phase contrast microscopy, was sufficient for us to ascertain gross differences in vesicle quantity and quality. We expect that further modification of the polymer solution drop-casting procedure, such as choice of solvent and temperature of rehydration, would eventually allow us to improve the yield and quality of polymer GUVs.

More importantly, drop-casting allowed us to prepare substrates for rehydration in glass Petri dishes, which could then be arrayed in 6-well plates to emulate a multi-well format. This allowed for a limited form of multi-parameter testing. Due to the limited availability of glass Petri dishes of smaller size, it was difficult to use plates with more wells. The use of glass was necessary since the chloroform in the lipid and polymer solutions would have corroded polystyrene multi-well plates. Of course, commercially available glass [48], glass-coated [49,50], and polypropylene [51] well plates would also withstand exposure to the solvent. However, these tend to be costly, rare, and restricted to the 96-well format. An incidental alternative that presented itself in this work is the custom 3D printing of multi-well plates using polypropylene as the printing material. Indeed, the webpage www.thingiverse.com (accessed on 3 January 2024) offers custom multi-well plates designed for those who would like to explore this avenue of solutions. We offer two additional solutions: (i) coating the walls of the wells with the hydrogel, as well as (ii) the gradual dropwise addition and evaporation of the lipid or polymer solution on the pre-hydrogel thin film, in order to confine the solvent to the thin film and avoid contact with the polystyrene. We have shown how these two simple techniques have allowed us to cast hydrogels and other substrates into conventional cell culture multi-well plates for GUV production. Not only did this allow us to handle multiple samples simultaneously, but it also allowed the process to be down-scaled in volume and up-scaled in number to facilitate multi-parameter testing, or up-scaled in volume and down-scaled in number for increased GUV yield, by merely using plates of different well numbers. The ability to use a smaller volume for preliminary testing is particularly useful when the test material is scarce or expensive.

Armed with this protocol, we then explored the use of dried films comprising low-gelling point agarose and polyvinyl alcohol, as well as combinations of both, for GUV formation. This allowed us to establish standards for later comparison using the previously reported materials, and offered us a chance to determine if mixing the two would offer advantages such as improved yield and reduced agarose contamination. We have shown how a 1:1 mixture of agarose and polyvinyl alcohol appears to improve the yield of GUVs, although we have not ruled out possible contamination of the vesicles with agarose or polyvinyl alcohol, a side effect that has been amply reported [32,33]. They also readily produced GUVs of comparable yield and quality from lipids extracted from the B-lymphoblastoid cell line, TF228.1.16, although less so from the T-lymphoblastoid cell line, A3R5.7. Interestingly, the hydrogels that are left after harvesting the GUVs can be processed and re-dried for continued GUV production, at least twice. We expect that further reuse would be possible until wear and tear of the hydrogels makes it impractical. This would be useful in cases where material is scarce or time is limited, or when certain assay parameters, such as substrate material, have to be kept constant. Reuse also serves to flush free agarose or PVA from the matrices, thereby reducing potential contamination of the GUVs. However, this might also have led to the reduced yield, since partial dissolution of the agarose was proposed by Horger to facilitate GUV production [29]. Curiously, we did not suffer from delamination of the hydrogels except in rare, irregular cases, despite not having treated the surfaces for improved hydrogel adhesion. We did, however, observe cracking when the hydrogels were cast in 24-well plates, necessitating a second coat of the hydrogel. We also show that the conditions used are mild enough, despite the elevated temperature, to enable the encapsulation and function retention of porcine liver esterase. This allowed the resultant GUVs to be labelled using calcein-AM, just as cells are for cytometry and other fluorescence-based analyses [52,53]. To our knowledge, this is the first demonstration of such a labelling method for GUVs, and it reiterates the potential of GUVs as cell metabolism models. The fact that calcein-AM now has commercially available derivatives offering wide-ranging fluorescence profiles [54] means that esterase-containing GUVs of different types, perhaps carrying different cargo or treated differently in multiplexed samples, can be differentially labelled and tracked. Other acetoxymethylated probes, such as Indo-1AM and Fura-2AM for calcium tracking, can potentially be used as well, thus extending the usefulness of PLE-functionalised GUVs.

Ultimately, however, our interest was in using our multi-well protocol to evaluate the GUV-producing behaviour of diverse substrates emulating agarose and PVA. We wanted to ascertain if any material, dried or otherwise, that can support a thin film of amphiphile and is then able to absorb water and swell, could be used for GUV production. There exists a plethora of hydrogels used in biomedical technology, some of which we suspected would behave as desired [55]. Many of these, including cross-linked matrices comprising hyaluronic acid [56,57], collagen [57], poly(ethylene glycol) diacrylate–polyurethane [58], and Matrigel [59,60,61], among others, mimic extracellular matrix material (ECM) and are used to support the attachment of cells and coax them into the appropriate behaviour. By their nature, they are able to imbibe water and often change shape or size according to environmental conditions [55]. Such hydrogels were ready options for testing our idea. We chose PEG-DA as an example of a synthetic polymer material [62,63], and hyaluronic acid and Matrigel to exemplify natural polymers found in ECM. DNA hydrogels have also come to be explored as potential matrices for drug encapsulation and controlled release [64,65,66]. The idea of a DNA-based substrate for liposome formation is the intriguing reverse of the more common effort to encapsulate DNA in liposomes. For our purpose, we selected salmon DNA, which has been extensively studied for the production of responsive drug-infused hydrogels [64,67,68,69,70]. Just as for PEG-PLA, we hoped to demonstrate that well-characterised, biocompatible, and well-established systems such as these may already present an available pool of test materials, and that it is not imperative to seek exotic reagents. All were also chosen because of their commercial ubiquity, making them easily available.

The use of multi-well plates facilitated our investigations considerably by allowing us to simultaneously test diverse formulations. PEG-DA readily formed hydrogels, although the yield of GUVs from their use was less than expected. This might have been due to the relative stiffness and therefore reduced swellability of PEG-DA hydrogels. To our surprise, irradiating the hyaluronic acid and Irgacure from the PhotoHA^®^-IRG, Methacrylated Hyaluronic Acid Hydrogel Kit did not result in the expected hydrogel. Nonetheless, some crosslinking of the hyaluronic acid must have occurred in at least the formulations comprising 1% hyaluronic acid supplemented with 10%, 30%, and 100% Irgacure, since hydrogel material was left after exposure to rehydration buffer. Oxygen is known to reduce the efficiency of radical-induced crosslinking by reacting with the activated chain ends and causing chain termination, especially during hydrogel formation [71]. We therefore expect that depleting the reaction mixtures’ oxygen or displacing it with nitrogen would improve hydrogel formation. However, the references we consulted, including those of the vendor, do not include such steps in their protocol. Another factor that might affect hydrogel formation is elevated temperatures. Hyaluronic acid is a mammalian ECM linear polysaccharide of the glycosaminoglycans family, comprising D-glucuronic acid and N-acetyl-d-glucosamine, cross-linked in alternation via 1,3 and 1,4 glycosidic bonds [72]. Its thermal stability in both powder and solution form has been extensively studied, yielding results of crucial importance to biomedical applications [72,73,74,75,76]. Exposure to elevated temperatures is known to cause a reduction in the molecular weight of hyaluronic acid chains, although the extent to which this occurs is dependent on the initial molecular weight of the material used, whether it is in the solid state or in solution, the exposure temperature, and the duration of exposure. The results reported by Mondek et al. [72] suggested that a solution of 1.67 MDa hyaluronic acid exposed to 60 °C for up to 12 h would lose approximately 15% of its molecular weight. In our case, following crosslinking, our hyaluronic acid of a molecular weight of 100–150 kDa had been exposed to 50 °C for 3–14 h. It is possible that this similarly led to the degradation of our material, resulting in the absence of a visible hydrogel following dehydration.

We were confronted with a similar difficulty with the salmon DNA from Sigma-Aldrich (D1626). When what was initially described by the vendor as DNA fragments of 2000 bp were used to prepare 9% (*w*/*v*) DNA in NaBr solution, the mixture invariably produced a hydrogel by the mere process of mixing and shaking. The addition of water to these masses and incubation overnight at room temperature resulted in complete dissolution. As DNA hydrogel formation has been copiously reported for fragments of this size [64,65,69,77], we suspected that the fault lay instead with the DNA we had used. Indeed, when our reagent was characterised by both electrophoresis and a Genomic DNA Screen Tape Assay, we discovered that our DNA contained fragments of more than 20,000 bp, a difference of more than 10 times what was initially expected (Figure S22). Users whose work depend on the molecular weight of this product might wish to take note of this discrepancy. This would explain the difficulty we had in producing a 9% (*w*/*v*) solution from it. Nonetheless, using reduced concentrations, we were still able to produce hydrogels and amorphous gels that appeared to support GUV production. Of note is the fact that these materials did not have the mechanical consistencies of agarose, polyvinyl alcohol, or even PEG-DA hydrogels, regardless of the method of crosslinking used. In fact, there appeared to be mass loss after rehydration, which we attribute to insufficient crosslinking by both chemical curing and photocuring. We wonder if this shares a common cause with our problem of ineffective PhotoHA^®^-IRG crosslinking.

To summarise, each of the four biomedical hydrogels we tested presented unique physical characteristics including stiffness and tensile strength. Thanks to our approach of casting them in dishes and wells, this did not present difficulties in their handling. However, their structural properties might lead to unique challenges for novices in the following ways: (i) Hydrogels that cure relatively slowly, such as Matrigel and salmon DNA, risk running off the walls of wells before they solidify, thus compromising their ability to coat and protect polystyrene multi-wells against corrosion by chloroform. (ii) During dehydration, less rigid forms of hyaluronic acid and DNA hydrogels might collapse so severely that the resultant aggregates may not be able to rehydrate and swell. (iii) Matrigel is an extract comprising laminin, collagen IV, heparan sulfate proteoglycans, and entactin or nidogen, derived from the basement membranes of the Engelbreth-Holm-Swarm (EHS) mouse sarcoma [78,79,80]. As the amphiphiles are typically dissolved in chloroform or other organic solvents, the proteinaceous components of Matrigel might denature, or perhaps form contaminating aggregates from exposure. As such, care should be taken to avoid prolonged contact with these solvents. (iv) Rehydration of membrane lipids and amphiphilic copolymers typically proceeds at a temperature above that of their phase transition [81]. These temperatures tend to be elevated, thus making materials like hyaluronic acid vulnerable to thermal degradation, as discussed above. (v) Finally, the rigidity of typical PEG-DA hydrogel formulations might reduce their swellability and impede GUV formation.

We note, however, that although the GUV yields and quality may often be limited, every hydrogel species or amorphous gel that we have tested so far appears to be able to support giant unilamellar vesicle production. The performance of Matrigel hydrogels in particular exceeded our expectations. Unlike hyaluronic acid, it is a more complex ECM-like environment, held together by both covalent as well as non-covalent bonds [78], and possesses excellent hydration properties that might have helped it to facilitate GUV formation. Our observations emphasise the robustness of hydrogel-assisted GUV production, and demonstrate that improvements to this method, as well as the expansion of the range of materials available, are possible.

It should be remembered that the formation of GUVs is also affected by the interaction of the lipids and polymers with the hydrogel or substrate material [29,35,37]. Shielding the charged or polar regions of the amphiphiles through amphiphile–hydrogel interactions would impede hydration, and hence hinder the swelling and separation of the amphiphile layers. This would invariably reduce the yield of GUVs. Indeed, this appears to have been the case with the DNA-based hydrogels we tested. Our failure to produce sturdy hydrogels from salmon DNA, and their subsequent leaching, recalls the problem of agarose and polyvinyl alcohol matrix material contaminating GUV membranes. We have shown that substrate DNA material had in fact leached and been encapsulated by the GUVs in some cases. This might have been facilitated by Fickian diffusion of phospholipids into the DNA substrate and the mixing of the two materials. It is interesting to note that plasmids have also been reported to be capable of hydrogel formation [70]. Would a leachable hydrogel made of plasmids enable encapsulation of complete genetic devices (as defined in synthetic biology) into GUVs? While this leaching may be undesirable for typical applications, it does raise interesting possibilities for gene delivery and artificial cell construction. Although we had not probed any of the other hydrogels for substrate leaching, it is possible that PEG-DA, HA, and Matrigel might also have contaminated their respective GUVs. However, whether this would be undesirable would depend on the nature of the contamination (e.g., intra-lumenal or intra-membrane), and their biocompatible nature would make any adverse reaction from contact with their GUVs unlikely.

We expect the yield and quality of GUVs to be improved by optimising the substrate composition, curing time, and drying time. Conditions for substrate rehydration, especially the temperature and ionic strength of the buffer used, are heavily influenced by the physicochemical nature of the amphiphile [29] and can also be tweaked. Furthermore, it is likely that the mechanism of GUV production is different for the various substrates. It would be interesting to evaluate the performance of stimuli-sensitive hydrogels, whose physical characteristics, such as swellability, are sensitive to small changes in the external environment and can change accordingly [55]. Perhaps a phospholipid-coated stimuli-sensitive hydrogel implant can be induced to produce GUVs in situ when necessary, or a similar system deployed outdoors might be induced to do so by environmental perturbations. Clearly, there is much that can be defined and fine-tuned. Our adaptation of the hydrogel-assisted GUV production protocol for the multi-well format will facilitate these studies tremendously.

Nonetheless, our observations demonstrate the effectiveness of the hydrogel-assisted method in producing cell-sized vesicles. They have given us a new perspective on the formative power of simply providing alternating dry and wet conditions. It leads us to wonder if similar natural materials in the biotic environment, such as bacterial biofilms, ECM, and other carbohydrate masses, might also behave similarly under similar conditions. Extreme swings in weather might provide the dry–wet cycles necessary for creating hydrogel and amorphous gel systems from these substances. Consider also that amphiphilic material, including biotic phospholipids, might be deposited onto them during a dry phase, and be rehydrated to form GUVs in a subsequent wet phase. Could this lead to the occasional spontaneous de novo emergence of novel protocells? Indeed, this is posited to have happened and given rise to the first protocells in the prebiotic Hadean environment, especially at geothermal vents and hot springs, which experienced such dry–wet cycles [82,83]. Could this happen in an extra-terrestrial setting [82]?

Through our work, we hope to demonstrate the robustness of hydrogel-assisted GUV production and show that with our approach, one can quickly screen candidate materials for their ability to support this process. We also show that a rich pool of such candidate materials already exists, some of which may offer even more desirable characteristics and abilities, such as biocompatibility and tunability, that could cater to diverse research needs. We hope that these resources will encourage deeper exploration into the use of GUVs, particularly in studies in areas such as biosensor development, artificial cell research, origin of life, protocell evolution, and perhaps even exobiology. We also hope to encourage novices into these disciplines, who might otherwise have concerns about the difficulties and limitations of producing GUVs.

## 3. Conclusions

To summarise, we have developed a modified protocol for hydrogel-assisted giant unilamellar vesicle production that allows it to be used in a multi-well format. To validate this protocol, we successfully produced GUVs from DOPC, DPPC, BdEO950, and PEG-PLA, as well as total lipids extracted from two different cell lines, using AGA, PVA, and AGA-PVA hydrogels. We then used it to show that AGA-PVA 1:1, PEG-DA, cross-linked hyaluronic acid, Matrigel, and even DNA hydrogels and amorphous gels can be used to produce GUVs. It also allowed us to show that used AGA, PVA, and AGA-PVA hydrogels can be reused following appropriate treatment. We further demonstrated the utility of our approach by encapsulating porcine liver esterase in GUVs and developing a novel means of labelling GUVs with calcein-AM for fluorescence analysis. Future work will focus on exploring these and other hydrogel systems, and especially on understanding their mechanism of GUV production. This would allow us to improve the yield and quality of GUVs produced and expand the repertoire of materials and techniques for GUV production.

## 4. Materials and Methods

Lipids and polymers: 1,2-dioleoyl-sn-glycero-3-phospho-choline (DOPC, SKU# 850375C, L/N 181PC-313), 1,2-dipalmitoyl-sn-glycero-3-phos-phocholine (DPPC, SKU# 850355C, L/N 160PC-319), 18:1 Liss Rhod PE (Rh-PE, SKU# 810150C, L/N 5340CHN090), and Cholesterol (SKU# 700000, L/N CH-50) were purchased from Avanti Polar Lipids (700 Industrial Park Drive, Alabaster, AL, USA). Poly (butadiene-b-ethylene oxide) 1,2 addition (BdEO950, Sample#: P41807C-BdEO) was obtained from Polymer Source, Inc. (124 Avro avenue, Dorval (Montreal), QC, Canada). Poly(ethylene glycol)-block-polylactide methyl ether (PEG-PLA, 659665, L/N MKCS5866) was supplied by Sigma-Aldrich (Burlington, MA, USA).

Hydrogels and amorphous gels: Polyvinyl alcohol (PVA, SKU# 10849-250G, L/N BCBX4678), low-molecular-weight DNA (LMW, 31149, L/N BCCL1729) and high-molecular-weight DNA (HMW, D1626, L/N 249186) from salmon, poly(ethylenglycol) diglycidyl ether (PEGDGE, 475696, L/N MKCW1178), poly(ethylene glycol) diacrylate (PEG-DA, 437441, L/N MKCF5269), 2,2-dimethoxy-2-phenylacetophenone (DMAP, 196118, L/N MKCF8620), and ultra-low gelling temperature agarose Type IX-A (SKU# A2576-5G, L/N SLBP7931V), were purchased from Sigma-Aldrich, St. Louis, MO, USA. GelPilot^®^ LE Agarose (1062631, L/N 136258626) was from QIAGEN (Hilden, Germany). Merck (Darmstadt, Germany) supplied the PhotoHA-IRG^®^, Methacrylated Hyaluronic Acid Hydrogel Kit (CC326-2, L/N 411435 and Irgacure CC326-1, L/N 4119858), as well as the Corning^®^ Matrigel^®^ Basement Membrane Matrix (CLS354234, L/N 21623002).

Stains: Calcein-AM (SKU# 17783, L/N BCCB5368) was purchased from Sigma (Burlington, MA, USA), while Calcein Red™ AM (219, L/N 1331002) and Calcein Blue AM (22007, L/N 0971302) were from AAT Bioquest (5775 W Las Positas Blvd., Pleasanton, California, USA). Nile Blue A Oxazone (Nile Red, SKU# N-3013, L/N 47H3445) was from Sigma, USA, while Hoechst 33342 (62249, L/N UK2884032) was purchased from Thermo Fisher (Waltham, MA, USA).

The enzyme, porcine liver esterase (PLE, 46058, L/N BCCJ5320), was purchased from Sigma-Aldrich, USA, while the dimethyl sulphoxide (DMSO, SKU# 1029311000, L/N BCBM4408V) was purchased from Merck, Germany. The SYLGARD™ 184 Silicone Elastomer Kit (PDMS, GMID 01673921, L/N H052I4K182) was obtained from Dow (Midland, MI, USA), while tetramethylethylenediamine (TEMED, 161-0800, L/N Control 210012017) was from Bio-Rad (Hercules, CA, USA). The cell lines, A3R5.7 (ARP-12386) [84] and TF228.1.16 (ARP-11481) [85] were both from NIH HIV Reagent Program (formerly NIH AIDS Reagent Program) (managed by ATCC, Manassas, VI, USA). Please see Acknowledgements for detailed source information.

BRAND^®^ glass Petri dishes (BR455701) were purchased from Merck, Germany. Only the dishes (3 cm internal diameter), not the lids, were used.

### 4.1. Cell Culture and Total Lipid Extraction

Both A3R5.7 and TF228.1.16 were cultured in suspension in a Heraeus CO_2_ incubator at 37 °C, in a humidified atmosphere supplemented with 5% CO_2_. Both cell lines were cultured in a growth medium comprising basal medium supplemented with 10% fetal bovine serum (Gibco, Carlsbad, CA, USA), 2 mM L-glutamine (Gibco, USA), and 1 mg/mL geneticin (Gibco, USA). Roswell Park Memorial Institute medium (RPMI) and Dulbecco’s Modified Eagle’s Medium (DMEM) were used as the basal medium for A3R5.7 and TF228.1.16, respectively. To prepare lipid extracts for use in GUV production, 10^7^ cells were pelleted by centrifugation at 200× *g* for 5 min and discarding the supernatant. The pellet was then thoroughly resuspended in 1 mL of Dulbecco’s phosphate-buffered saline (DPBS). The cell suspension was then vortex-mixed with 1 mL of methanol, followed by 1 mL of chloroform. The resultant suspension was then ultrasonicated using the Ultrasonic Processor UP200St with the Sonotrode S26d2, at settings of 50% power and 100% amplitude. Sonication was performed five times, each for 30 s followed by a rest period of 30 s. The sample was then incubated at room temperature for 30 min, and then at 4 °C overnight. Subsequently, the mixture was vortex-mixed, then centrifuged at 14,100× *g* for 10 min. The clear liquid phase under the pellet (≈500 µL) was then carefully harvested, taking care to avoid collecting any of the clear upper phase. This lipid extract was then stored at −20 °C until used.

### 4.2. Preparation of Hydrogels and Amorphous Gels

Table S2 summarises all of the substates tested in this study.

#### 4.2.1. Agarose (AGA) and Polyvinyl Alcohol (PVA) Hydrogels

Precursor solutions of 1% (*w*/*v*) agarose Type IX-A and 5% (*w*/*v*) PVA were prepared in ultrapure water. Agarose powder was added to ultrapure water and carefully microwaved until dissolution, while the PVA powder in ultrapure water was autoclaved at 121 °C for 20 min, then shaken at 200 rpm on an orbital shaker until complete dissolution. The precursor solutions were brought to 50 °C before being dispensed into similarly preheated containers. For casting in glass Petri dishes of 3.4 cm internal diameter, 1 mL of precursor solution was dispensed into each dish and immediately dried at 50 °C in a convection oven overnight. For 96-well plates, 100 µL of precursor was added to each well instead. Casting in 24-well plates required 300 µL of precursor per well, which was then dried at 50 °C for 60 min, followed by the overlay of an additional 300 µL of precursor, which was also dried at 50 °C for 60 min. The overlay was necessary in order to fill in the cracks, which appeared in the first layer after drying. When polystyrene multi-well plates were used, care was taken to swirl the plates, in order to coat the walls of the wells with precursor solution prior to oven drying. This wall-coating procedure was also used for all formulations of hydrogels and amorphous gels described below. AGA-PVA hydrogels were prepared from precursor solutions comprising agarose and PVA mixed at the appropriate volumetric ratios. Prior to reuse, rehydrated AGA, PVA, and AGA-PVA hydrogels were submerged in 1 mL of ultrapure water, then incubated at 50 °C in the oven for 30 min, with a lid to reduce loss to evaporation. The water was then used to carefully rinse the surface of the hydrogels before being removed. This washing step was repeated once. The hydrogels were then placed in the oven at 50 °C without a lid to allow for the complete evaporation of the residual water.

#### 4.2.2. Poly(ethylene Glycol) Diacrylate (PEG-DA) Hydrogels

For casting in 24-well plates, 600–1000 µL of PEG-DA was added to each well. DMAP at 0.09–0.56% (*w*/*w*) relative to the PEG-DA content was then dissolved in minimal ethanol and mixed with the PEG-DA. Ultrapure water was then added to bring the reaction volume to 1 mL. The precursor solutions were subsequently irradiated with a UV hand lamp (Herolab NU-8, H468.1) at 365 nm for 5 min, then incubated at room temperature for 5 min to allow for the completion of the crosslinking reaction. The resultant hydrogels were rinsed twice with ultrapure water and once with ethanol, before being dried at 50 °C in a convection oven overnight.

#### 4.2.3. Hyaluronic Acid (HA) Hydrogels

Precursor solutions were prepared by shaking 1% methacrylated HA from PhotoHA-IRG^®^ in ultrapure water at 4 °C for 30 min, then adding 0.1–100% (*w*/*w*) of Irgacure in methanol relative to the HA content. This was then dispensed into 24-well plates (200 µL per well) or 96-well plates (100 µL per well) and irradiated at 365 nm for 15 min, followed by incubation at room temperature for 5 min. The resultant products in 24- and 96-well plates were then incubated at 50 °C for 3 and 14 h, respectively.

#### 4.2.4. Matrigel Hydrogels

All working steps prior to incubation at 37 °C were performed on ice, and all consumables were pre-cooled to 4 °C. Frozen Matrigel was thawed on ice at 4 °C overnight, then diluted to concentrations of 2, 3, and 4 mg/mL in ultrapure water or 1× DPBS. 200 µL of the diluted Matrigel solutions was added to wells of a 24-well plate, while 100 µL and 200 µL of undiluted Matrigel stock solution at 9.7 mg/mL was used. The solutions were evenly spread over the bottom of the wells using micropipette tips, with care taken to also coat the sides of the wells. Then, the plate was incubated with the lid closed at 37 °C for 30 min to allow the Matrigel to solidify. After incubation the lid was removed, and the hydrogels were dried at 50 °C overnight.

#### 4.2.5. DNA Hydrogels and Substrates

Low-molecular-weight (LMW) salmon sperm DNA was dissolved in 4 mM sodium bromide to concentrations of 5%, 10%, and 20% (*w*/*v*) at room temperature overnight. High-molecular-weight (HMW) salmon sperm DNA was used at 1% or 2. 5% (*w*/*v*) instead. To prepare single-stranded LMW-DNA precursors, the DNA was heated to 90 °C for 10 min. For chemical crosslinking, PEGDGE was added at 5–100% (*w*/*w*) relative to the DNA content, and the pH was adjusted to 10 using TEMED. The mixture was then covered and incubated in the oven at 50 °C for 3 h, before drying at 50 °C overnight. For photoinitiation of crosslinking, Irgacure dissolved in methanol was added at 0.1–1% (*w*/*w*) relative to the DNA content. The reaction mix was then irradiated at 365 nm for 15 min, incubated at room temperature for 5 min, and then dried at 50 °C overnight. For 24- and 96-well plates, 1 mL and 100 µL of precursor solution was used, respectively.

### 4.3. Preparation of Lipid and Polymer Solutions

Membrane-forming lipid or polymer solutions were prepared from stock chloroform solutions of DOPC, DPPC, BdEO950, PEG-PLA, and cholesterol. Different formulations were prepared by mixing the appropriate components in the desired molar ratio, then adjusting the volume to bring them to the desired concentration. The final concentration of DOPC, DPPC, BdEO950, and PEG-PLA was fixed at 5.1 mM for all formulations. In formulations for producing fluorescent DOPC-containing GUVs, Rh-PE was added to a final concentration of 0.1 mol% relative to DOPC. Total lipid extracts were used as prepared. Table S3 summarises all of the amphiphiles used in this study.

### 4.4. Production of Giant Unilamellar Vesicles by Reagent Spreading

To compare our drop-casting method of GUV production with the conventional method of vesicle production, the method described by Greene et al. (2016) [31] was followed. Briefly, a microscopy coverslip was coated with 300 µL of 1% LE Gelpilot^®^ agarose by spreading until solidification. The agarose was then dried in an oven at 40 °C for >60 min. Then, 30 µL of lipid or polymer solution was applied to the agarose coat and spread with the long edge of a syringe until the chloroform evaporated. The slide was then placed under vacuum at room temperature overnight, to ensure complete removal of the chloroform. A PDMS mould (see Figure S1 for details) with a 1 cm-diameter central hole was then placed on the coated agarose and the slide was transferred to a hotplate stirrer (MH 15, Rotilabo^®^, Roth) set to 40 °C. 500 µL of 0.6 Osm/kg sucrose was then dispensed into the PDMS well, which was then covered to prevent evaporation. Rehydration of the lipid and polymer films proceeded for 60 min. To harvest the GUVs, half the volume of the vesicle suspension was collected with a micropipette and used to gently rinse the agarose hydrogel, and the GUV suspension was then transferred to a fresh 1.5 mL centrifuge tube. To facilitate the production of PDMS moulds of consistent quality and dimensions, a negative mould was 3D printed and used.

### 4.5. Production of Giant Unilamellar Vesicles by Reagent Drop-Casting

The lipid or polymer solutions were applied dropwise to the hydrogel- or substrate-coated containers, using a glass syringe. For Petri dishes, 24-, and 96-well plates, 120 µL, 50 µL, and 10 µL of solution was used, respectively. After allowing the chloroform to evaporate at room temperature for 30 min, residual solvent was evaporated at 50 °C in a convection oven for 60 min. Subsequently, rehydration buffer comprising 0.6 Osm/kg sucrose in water, pre-warmed to 50 °C, was added to the coated hydrogels or substrates. For encapsulating porcine liver esterase (PLE), 46 U PLE per mL of 0.6 Osm/kg sucrose was used instead. The PLE solution was prepared by dissolving the enzyme crystals in the sucrose overnight at 4 °C with rotation. The preparation was then filtered through a 0.22 µm pore-sized syringe filter before being warmed to 50 °C and used for rehydration. For Petri dishes (3 cm internal diameter), 24-, and 96-well plates, 2 mL, 1 mL, and 200 µL of rehydration buffer was used, respectively. The samples were then covered and incubated at 50 °C for 60 min. To harvest the GUVs, half the volume of the vesicle suspension was collected with a micropipette and used to gently rinse the hydrogel or substrate to release any vesicles that may have adhered to the hydrogel. The GUV suspension was then transferred to a fresh 1.5 mL centrifuge tube and stored at 4 °C until used.

### 4.6. Fluorescence Labelling of GUVs

To prepare the GUVs for fluorescence labelling, 100 µL of either 0.6 Osm/kg D-mannitol or 2.5× Dulbecco’s Phosphate Buffered Saline (DPBS) without calcium or magnesium (low density exchange buffers) was carefully added to 100 µL of sample with gentle stirring and pipette mixing. The mixture was then centrifuged at 34× *g* for 10 min. Subsequently, 190 µL of supernatant was replaced with 190 µL of fresh exchange buffer and the pellet was gently resuspended. Centrifugation was repeated, and 190 µL of the supernatant was removed. This resulted in a 10-fold concentration of the GUV suspension (10×). If the GUV number density was to be maintained, then 90 µL of fresh exchange buffer was to be added. To label the GUV membranes, Nile Red in DMSO was added to a final concentration of 1 µg/mL. The mixture was then incubated at 37 °C for 60 min. For staining with calcein, calcein-AM, calcein-AM Red, or calcein-AM Blue was added to the sample at a final concentration of 5 µg/mL. The reactions were then incubated at 37 °C for 60 min. To label contaminant DNA in the samples, Hoechst 33342 was added to the GUVs at a final concentration of 3 µg/mL. The reactions were incubated at 37 °C for 30 min. All reactions were then purified of excess reactants by centrifugation and the extra GUV buffer was replaced with fresh exchange buffer.

### 4.7. Microscopy

A Nikon Eclipse Ts2R microscope equipped with standard DAPI (Ex 377/50 nm, Em 447/60 nm), GFP (Ex 472/30 nm, Em 520/35 nm), and TRITC (Ex 540/26 nm, Em 605/56 nm) filters was used for phase contrast and epi-fluorescence microscopy. *Gryphax* (version 1.1.8.153) was used to capture images. GUVs were visualised in 96- or 384-well plates with clear bottoms, by diluting 10 µL of sample with two volumes of either 0.6 Osm/kg mannitol or 2.5× DPBS. The GUVs were then allowed to sediment before microscopy. Labelled samples were interrogated with epi-fluorescence microscopy using the TRITC filter cube for Nile Red and calcein Red, the FITC filter cube for calcein, and the DAPI filter cube for calcein Blue and Hoechst stains. Z-stack fluorescence imaging was performed using a Leica DMI6000B operated using *Leica Application Suite Advanced Fluorescence* (*LAS AF*, version 2.6.3.8173) software. All micrographs shown are representatives of data from at least three repeated experiments, unless stated otherwise.

### 4.8. Osmometry

The osmolality of all buffers was determined by freezing point depression, using an Osmomat 030 cryoscopic osmometer. All buffers were adjusted to 0.6 Osm/kg using the respective solute and solvent, in order to ensure equiosmolar conditions for the GUVs. The osmolality of 2.5× DPBS was determined to be 0.6 Osm/kg.

### 4.9. Analytical Flow Cytometry

Analytical flow cytometry was performed using a BD FACSCanto II™ flow cytometer (Firmware version 1.4.7). The device was equipped with 3 lasers (488 nm, 633 nm, and 405 nm) and photomultiplier tube detectors for Pacific Blue, AmCyan, FITC, PE, APC, PerCP-Cy5.5, PE-Cy7, and APC-Cy7. The data were collected using the *BD FACSDiva* Software (version 6.1.3) and processed using *FlowJo* (version 10.10.0). Gating strategy: GUVs comprising DOPC and cholesterol in a 2:1 molar ratio (DC2:1) were analysed and the SSC-FSC scatter plots for 10,000 events were noted. The GUVs were also microscopically inspected to ensure that the contents were predominantly vesicular. The sample was then treated with 1 volume of DMSO and analysed again. DMSO caused the GUVs to collapse into smaller vesicles or dense particles [86,87,88,89]. This was verified using phase contrast microscopy. The region in the scatter plot encompassing events that were no longer observed after this treatment, due to the collapse of the GUVs, was manually area-gated and designated P1. All scatter plots shown are representatives of data from at least three repeated experiments, unless stated otherwise.

## Data Availability

The data presented in this study are openly available in Zenodo at doi:10.5281/zenodo.14378774.

## Supplementary Materials

The following supporting information can be downloaded at: https://www.mdpi.com/article/10.3390/gels11010029/s1, Table S1: Summary of selected reports describing hydrogel-assisted GUV production; Table S2: Summary of materials and their crosslinking agents used for producing substrates in our work; Table S3: Summary of lipids and block co-polymers used for producing GUVs in our work; Figure S1: Render showing design of poly(lactic acid) mould for casting PDMS mould; Figure S2: SSC-FSC scatter-plots of GUVs made from (a) DC1:0, (b) PC1:0, (c) DC2:1, (d) PC2:1, (e) DP1:0, and (f) PPC1:0 on AGA hydrogels; Figure S3: Phase contrast micrographs of GUVs produced from various membrane materials on PVA hydrogels; Figure S4: SSC-FSC scatter-plots of GUVs produced from (a) DC1:0, (b) PC1:0, (c) DC2:1, (d) PC2:1, (e) DP1:0, and (f) PPC1:0 on PVA hydrogels; Figure S5: Comparisons of (a) size distribution (FSC), (b) complexity distribution, and (c) SSC-FSC scatter-plots of GUVs made on PVA; Figure S6: Phase contrast micrographs showing DC2:1 and PC2:1 GUVs produced various hydrogels; Figure S7: Phase contrast micrographs of GUVs made from DC2:1 on (a) AGA-PVA 2:1, and (c) AGA-PVA 1:2, as well as PC2:1 on (b) AGA:PVA 2:1 and (d) AGA-PVA 1:2; Figure S8: SSC-FSC scatter-plots of GUVs made from DC2:1 on (a) AGA-PVA 2:1, (c) AGA-PVA 1:1, and (e) AGA-PVA 1:2, as well as PC2:1 on (b) AGA:PVA 2:1, (d) AGA-PVA 1:1 and (f) AGA-PVA 1:2; Figure S9: Graph showing yields of DC2:1 GUVs produced using (a) AGA, (b) PVA, and (c) AGA-PVA 1:1, as well as PC2:1 GUVs produced using (d) AGA, (e) PVA, and (f) AGA-PVA 1:1 hydrogels; Figure S10: Comparison of size (FSC) distribution of (a) DC2:1 and (b) PC2:1 GUVs and complexity (SSC) distribution of (c) DC2:1 and (d) PC2:1 GUVs produced using AGA, PVA, and AGA-PVA hydrogels; Figure S11: Comparisons of size distribution (FSC) of GUVs made from (a) DC2:1, and (b) PC2:1, as well as complexity distribution (SSC) of GUVs made from (c) DC2:1, and (d) PC2:1; Figure S12: Phase contrast micrographs showing DC2:1 and PC2:1 GUVs produced in polystyrene multi-well plates using various hydrogels; Figure S13: Phase contrast micrographs showing DC2:1 GUVs produced on various PEG-DA hydrogel compositions; Figure S14: Phase contrast micrographs showing DC2:1 GUVs produced on substrates made from HA and various concentrations of Irgacure; Figure S15: Phase contrast micrographs showing DC2:1 Rh-PE GUVs produced on Matrigel hydrogels at various concentrations; Figure S16: Phase contrast micrographs showing DC2:1 GUVs produced using various LMW DNA substrates; Figure S17: Phase contrast micrographs showing DC2:1 GUVs produced using various HMW DNA substrates; Figure S18: Micrographs showing DC2:1 + Rh-PE GUVs made from 10% LMW-dsDNA + 10% PEGDGE; Figure S19: Micrographs showing DC2:1 + Rh-PE GUVs produced on 2.5% HMW-DNA + 23% PEGDGE hydrogels; Figure S20: Graph showing GUV yields before and after reusing the AGA, PVA, and AGA-PVA 1:1 hydrogels; Figure S21: SSC-FSC scatter-plots showing GUVs produced from DC2:1 on AGA-PVA 1:1 (a) used for the first time, (c) reused once, and (e) reused twice, as well as GUVs produced from PC2:1 on AGA-PVA 1:1 (b) used for the first time, (d) reused once, and (f) reused twice; Figure S22: Genomic DNA Screen Tape Assay evaluation of DNA fragment lengths in Low molecular weight DNA (LMW-DNA) and high molecular weight DNA (HMW-DNA) from salmon.
