# Supplementary material for: Rapid Multi-Well Evaluation of Assorted Materials for Hydrogel-Assisted Giant Unilamellar Vesicle Production: Empowering Bottom-Up Synthetic Biology
Source: Gels. 2025 Jan 2;11(1):29. doi: 10.3390/gels11010029 (PMC11765364; doi:10.3390/gels11010029)
Supplement: Supplementary file 1 [file gels-11-00029-s001.zip › gels-3391401-supplementary.pdf]

Supplementary Materials

Rapid Multi-Well Evaluation of Assorted Materials for Hydrogel-Assisted Giant Unilamellar Vesicle Production: Empowering Bottom-Up Synthetic Biology

Cherng-Wen Darren Tan *, Magdalena Schöller and Eva-Kathrin Ehmoser

Institute of Synthetic Bioarchitectures, Department of Bionanosciences, University of Natural Resources and Life Sciences, Vienna, Muthgasse 11, Level 2, 1190 Vienna, Austria; magdalena.schoeller@boku.ac.at (M.S.); eva.ehmoser@boku.ac.at (E.-K.E.)

* Correspondence: darren.tan@boku.ac.at; Tel.: +43-1-47654-80457

Table S1. Summary of selected reports describing hydrogel-assisted GUV production. Abbreviations: 1,2-dioleoyl-sn-glycero-3-phosphocholine (DOPC); 1,2-dioleoyl-sn-glycero-3-phosphoglycerol (DOPG); 1,2-dioleoyl-sn-glycero-3-phospho-L-serine (DOPS); 1,2-dioleoyl-3-trimethylammonium-propane (DOTAP); 1,2-dipalmitoyl-sn-glycero-3-phosphocholine (DPPC); 1,2-dipalmitoyl-sn-glycero-3-phosphatidylethanol-amine-N- [methoxy(polyethylene glycol)-2000] (PEG-PE); 1-palmitoyl-2-oleoyl-sn-glycero-3-phosphatidylcholine (POPC); 1-stearoyl-2-oleoyl-sn-glycero-3-phosphocholine (SOPC); 1,2-dioleoyl-sn- glycero-3-phosphoethanolamine-N-(7-nitro-2-1,3-benzoxadiazol-4-yl) (ammonium salt) (18:1 NBD-PE); 1,2- dioleoyl-sn-glycero-3-phosphoethanolamine-N- [methoxy(polyethylene glycol)-2000] (ammonium salt) (PEG2000-PE); 1,2-dipalmitoyl-sn-glycero-3-phosphoethanolamineN-(cap biotinyl) (biotin-PE); 1,2-dipalmitoyl-sn-glycero-3-phosphoethanolamine-N- [methoxy(polyethyleneglycol)-2000] (DPPE-mPEG); Poly(ethylene glycol)-poly(ethyl ethylene) (PEG-PEE).

Hydrogel Material	Support	Method of casting precursor	Amphiphile	Method of casting amphiphile	Reference
Assorted agaroses<br><br><br><br>Polyacrylamide	Glass slide	Dip-coating<br>Supplemented by spreading with micropipette tip when needed<br><br>Sandwiched with glass slide	POPC<br>DOPS<br>POPG<br>PEG-PE<br>Asolectin from soybean<br>Cholesterol	Spread with rod or needle	Horger *et al.* (2009) [29]
Cross-linked detran(ethylene glycol)	Thiolated glass slide	Drop-casting	POPC<br>18:1 NBD-PE<br>PEG2000-PE<br>Cholesterol	Drop-casting	Mora *et al.* (2017) [34]
Polyvinyl alcohol	Vitrex-treated glass cover slips	Spreading	DPPC<br>DOPC<br>DOPG<br>DOPS<br>DOTAP<br>Cardiolipin<br>Biotin-PE<br>DPPE-mPEG	Spreading	Weinberger *et al.* (2013) [32]
Molecular biology agarose	Glass cover slip	Spreading with micropipette	Poly(ethylene glycol)-poly(butadiene) (PEG-PBD)<br>PEG-PBD-$NH3^{+}$<br>PEG-PBD -COO^{-}<br>PEG-PEE	Spreading with needle	Greene *et al.* (2016) [31]
Potato starch<br>Starch hydrolyzed grade	Borosil Petri dish	Spin-coating	SOPC<br>DOTAP<br>DOPS	Spin coating	Maoyafikuddin *et al.* (2020) [37]
Ultra-low gelling agarose<br><br>Polyacrylamide	Glutaraldehyde-functionalised glass coverslip	Spread using glass Drigalski spatula<br><br>Sandwiched between APTES-treated coverslip and glass slide	DOPC<br>POPC<br>DOPS<br>Cardiolipin	Spread using glass Drigalski spatula	Parigoris *et al.* (2020) [36]

*Table S2. Summary of materials and their crosslinking agents used for producing substrates in our work. Abbreviations used for the various formulations are indicated in parentheses under Abbreviations. *Ratios indicate volumetric ratios of AGA to PVA. **Numbers indicate the relative quantities of PEG-DA (P) and DMAP (D). See content for details.*

Hydrogels and amorphous gels:	*Abbreviation*	**AGA-PVA formulations*	***PD formulations*	*HA formulations*	*Matrigel formulations*	*DNA formulations*
Ultra-low gelling temperature agarose Type IX-A	AGA	AGA	PD60-15	1%HA + 10% Irgacure	2 mg/mL Matrigel-H_2O	10% LMW ssDNA + 5% PEGDGE
DNA gel agarose	-	PVA	PD60-37	1%HA + 30% Irgacure	2 mg/mL Matrigel-DPBS	10% LMW dsDNA + 5% PEGDGE
Polyvinyl alcohol	PVA	AGA-PVA 2:1	PD80-11	1% HA + 100% Irgacure	3 mg/mL Matrigel-H_2O	10% LMW ssDNA + 10% PEGDGE
Poly(ethylene glycol) diacrylate	PEG-DA (P)	AGA-PVA 1:1	PD80-28		3 mg/mL Matrigel-DPBS	10% LMW dsDNA + 5% PEGDGE
2,2-dimethoxy-2-phenylacetophenone	DMAP (D)	AGA-PVA 1:2	PD100-09		4 mg/mL Matrigel-H_2O	10% LMW dsDNA + 0.1% Irgacure
Hyaluronic acid	HA		PD100-22		4 mg/mL Matrigel-DPBS	10% LMW dsDNA + 1% Irgacure
Irgacure	-				Undiluted Matrigel	2.5% HMW DNA + 11.5% PEGDGE
Corning® Matrigel® Basement Membrane Matrix	Matrigel					2.5% HMW DNA + 23% PEGDGE
Low molecular weight salmon single-stranded DNA	LMW ssDNA					2.5% HMW DNA + 34.5% PEGDGE
Low molecular weight salmon double-stranded DNA	LMW dsDNA					10% LMW (ss/ds)DNA + 10% PEGDE
High molecular weight salmon DNA	HMW DNA					2.5 HMW DNA + 23% PEGDA
Poly(ethylenglycol) diglycidyl ether	PEGDGE					

Table S3. Summary of lipids and block co-polymers used for producing GUVs in our work. Abbreviations used for the formulations are indicated in parentheses under Abbreviations. Ratios indicate molar ratios of the preceding components.

Lipids and polymers:	*Abbreviation*	*Lipid formulations*	Polymer formulations
1,2-dioleoyl-sn-glycero-3-phospho-choline	DOPC (D)	DC1:0	PPC1:0
1,2-dipalmitoyl-sn-glycero-3-phos-phocholine	DPPC (DP)	DC2:1	PC1:0
1,2-dioleoyl-sn-glycero-3-phosphoethanolamine-N-(lissamine rhodamine B sulfonyl) (ammonium salt)	Rh-PE	DC2:1-PLE (porcine liver esterase)	PC2:1
Poly (butadiene-b-ethylene oxide) 1,2 addition	BdEO0950 (P)	DC2:1 + Rh-PE	–
Poly(ethylene glycol)-block-polylactide methyl ether	PEG-PLA (PP)	DP1:0	–
Cholesterol	(C)	–	–
Total lipid extracts from A3R5.7 and TF228.1.16	–	–	–

PDMS mould fabrication

The design for the 3D-printed negative mould for casting the PDMS was created in Autodesk Fusion 360. The filament material chosen for printing was poly(lactic acid) (Extrudr|FD3D GmbH, Austria), and the printer used was a Prusa MK3 Fused Deposition Modelling printer.

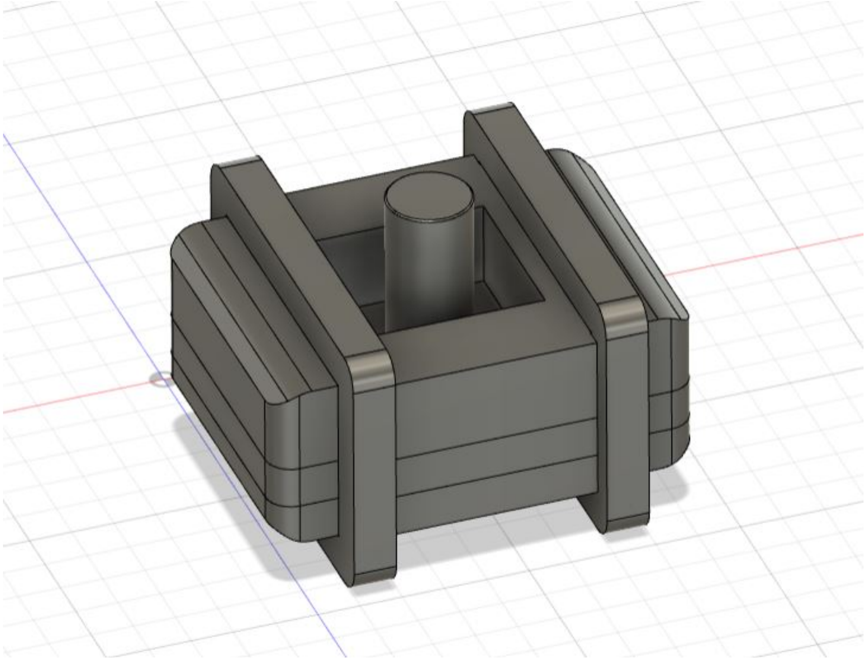

Figure S1. Render showing design of poly(lactic acid) mould for casting PDMS mould. Files for 3D printing are available upon request.

The PDMS was prepared by adding 1 part of curing agent to 10 parts of base and mixing thoroughly. The mixture was degassed prior to casting and after casting. After drying at 70 °C in a convection oven for 60 min, the solidified PDMS mould was removed from the 3D-printed negative.

Flow cytometry analysis of diverse GUVs produced on AGA hydrogels

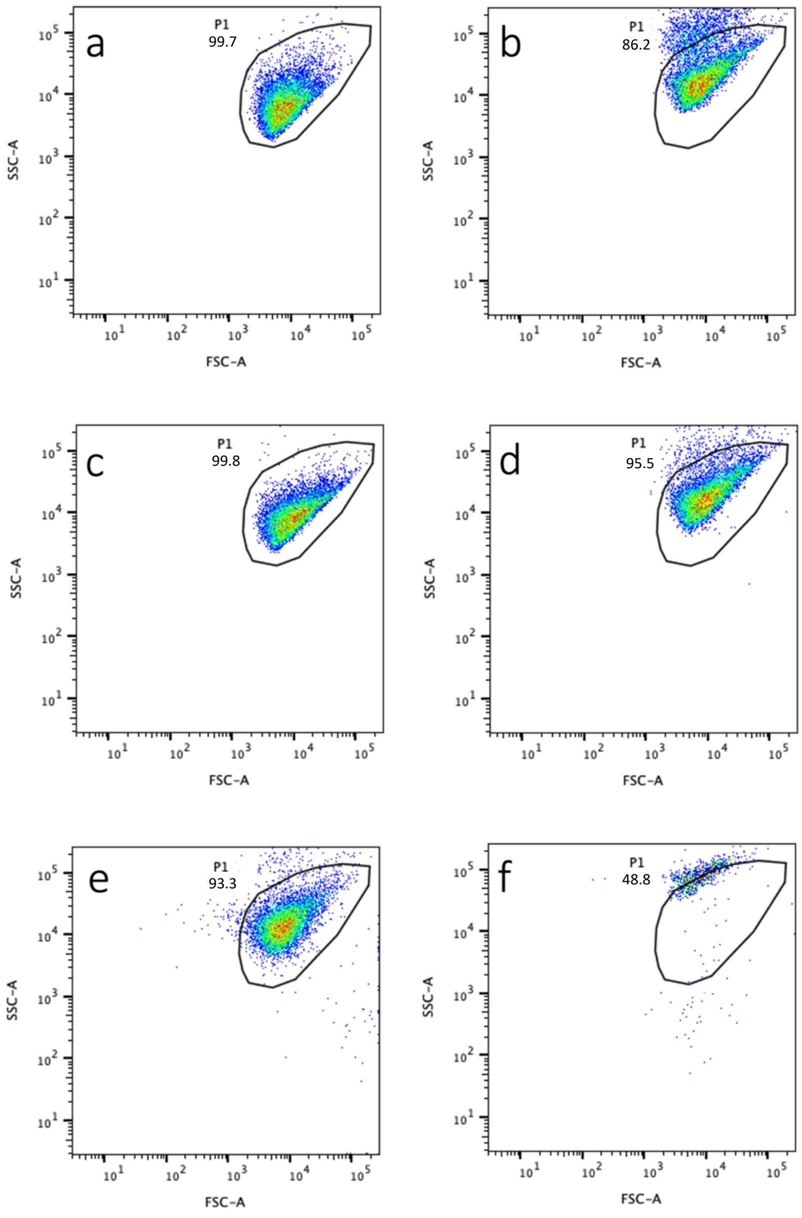

Figure S2. SSC-FSC scatter-plots of GUVs made from (a) DC1:0, (b) PC1:0, (c) DC2:1, (d) PC2:1, (e) DP1:0, and (f) PPC1:0 on AGA hydrogels. See Methods for gating strategy.

Evaluation of diverse membrane-forming materials on PVA hydrogels

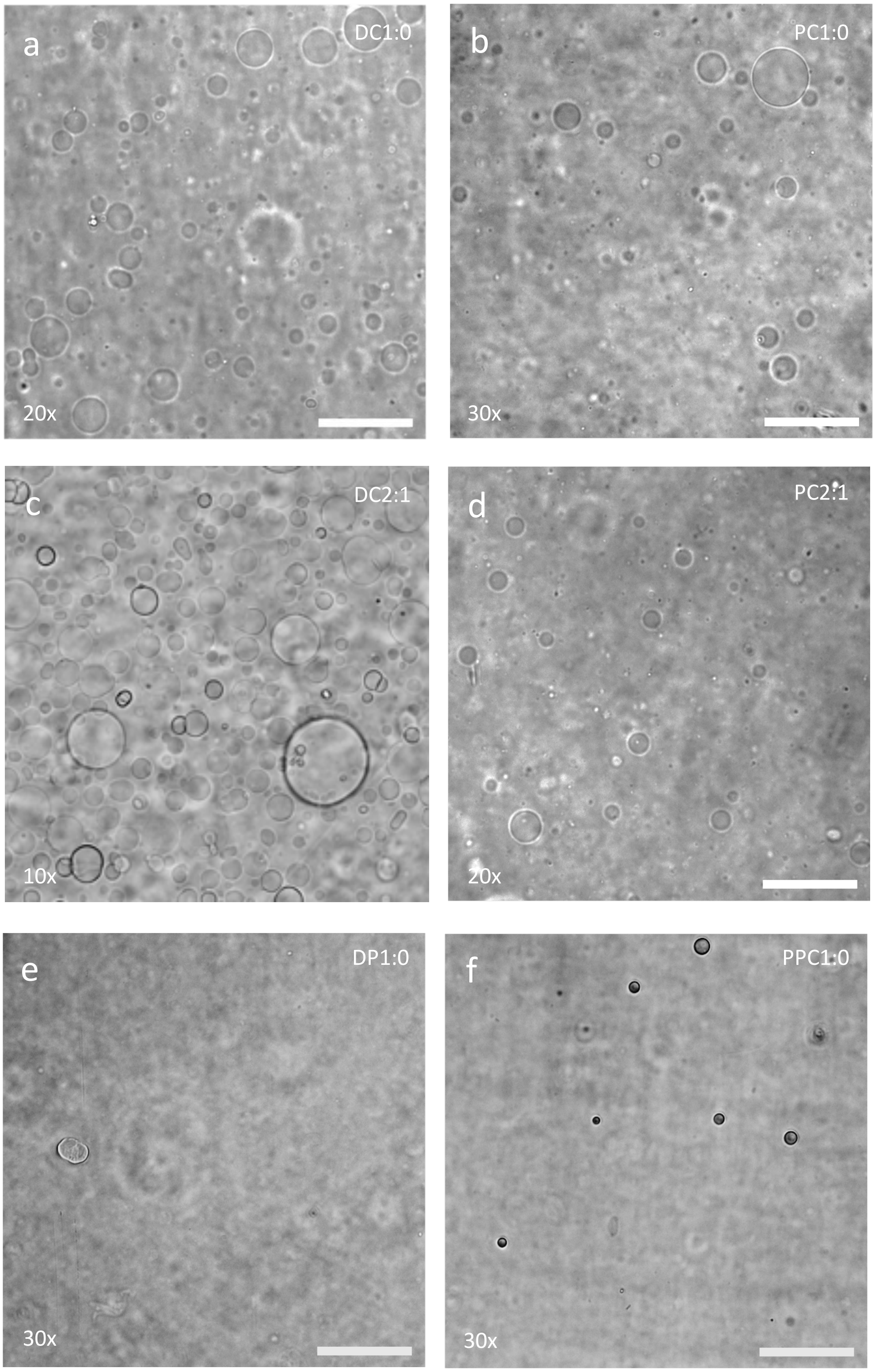

Figure S3. Phase contrast micrographs of GUVs produced from various membrane materials on PVA hydrogels. Factors (x) indicate how many times the GUV samples have been concentrated. Scale bar = 50 µm.

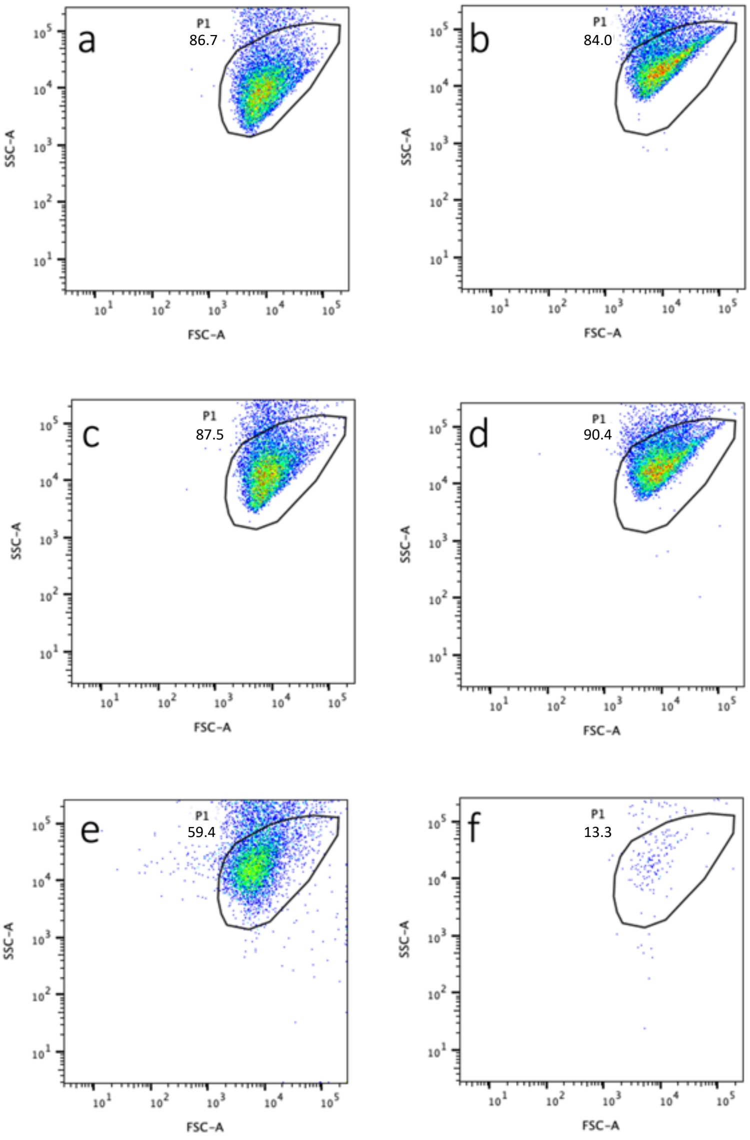

Figure S4. SSC-FSC scatter-plots of GUVs produced from (a) DC1:0, (b) PC1:0, (c) DC2:1, (d) PC2:1, (e) DP1:0, and (f) PPC1:0 on PVA hydrogels.

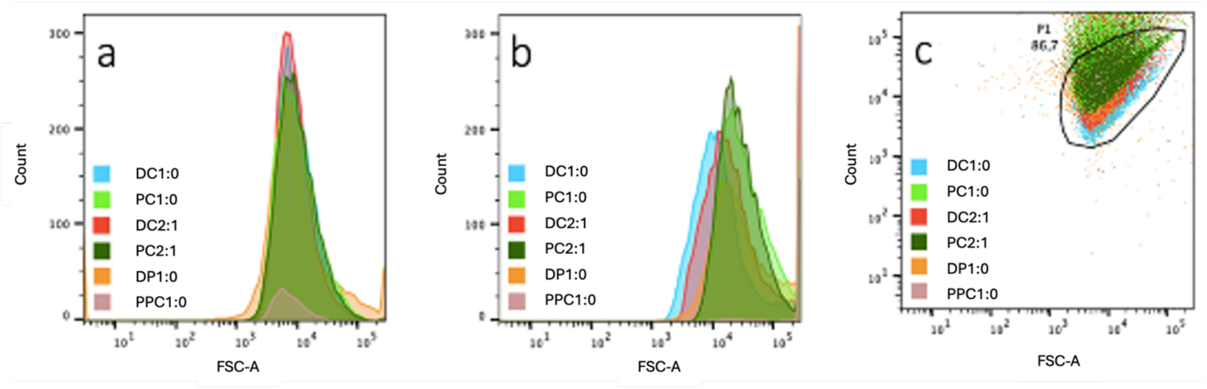

Figure S5. Comparisons of (a) size distribution (FSC), (b) complexity distribution, and (c) SSC-FSC scatter-plots of GUVs made on PVA.

DC2:1 and PC2:1 GUVs produced on AGA, PVA, and AGA-PVA hydrogels

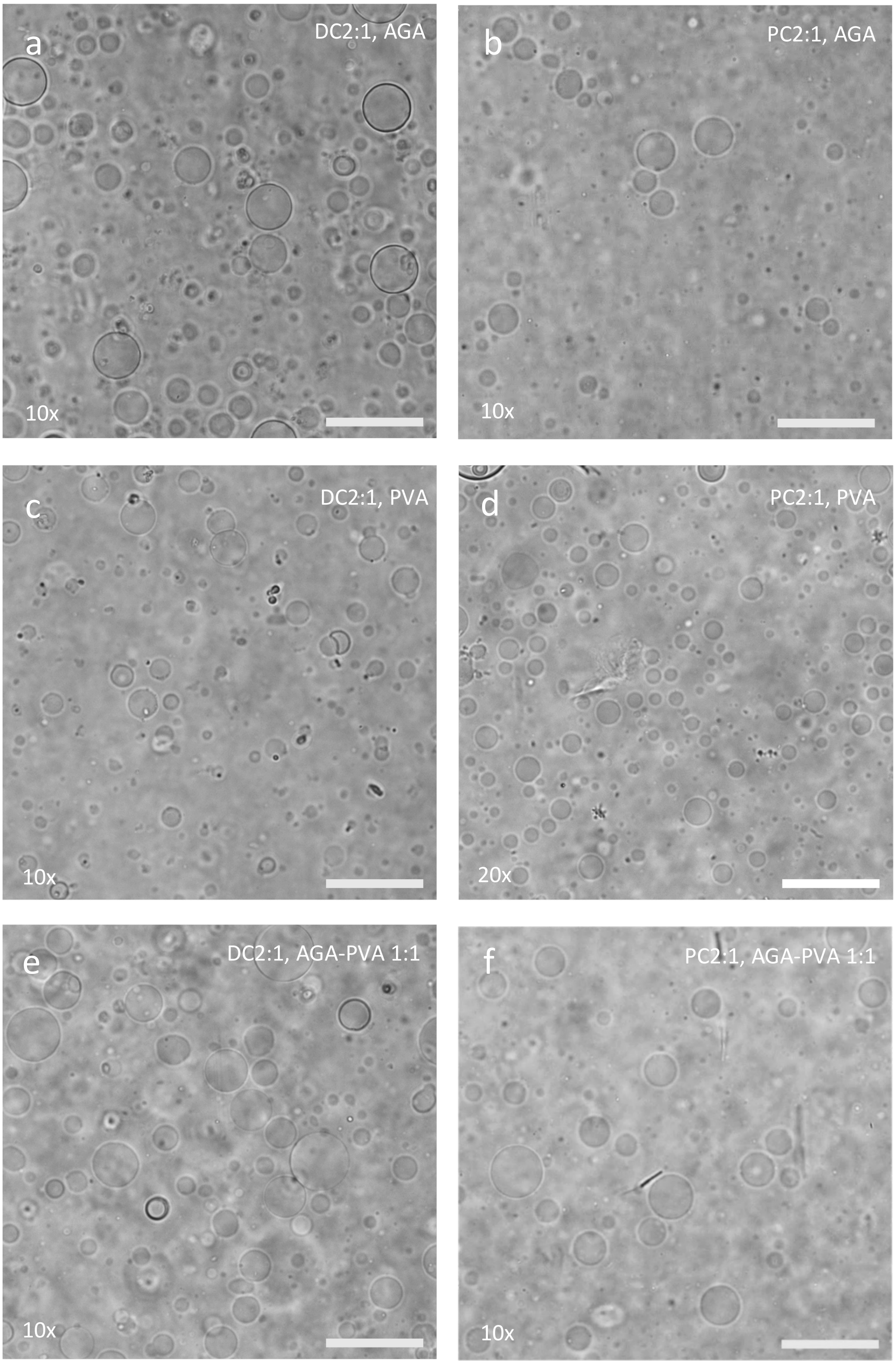

Figure S6. Phase contrast micrographs showing DC2:1 and PC2:1 GUVs produced various hydrogels. See Figure S7 for micrographs from the other formulations. Factors (x) indicate how many times the GUV samples have been concentrated. Scale bar = 50 µm.

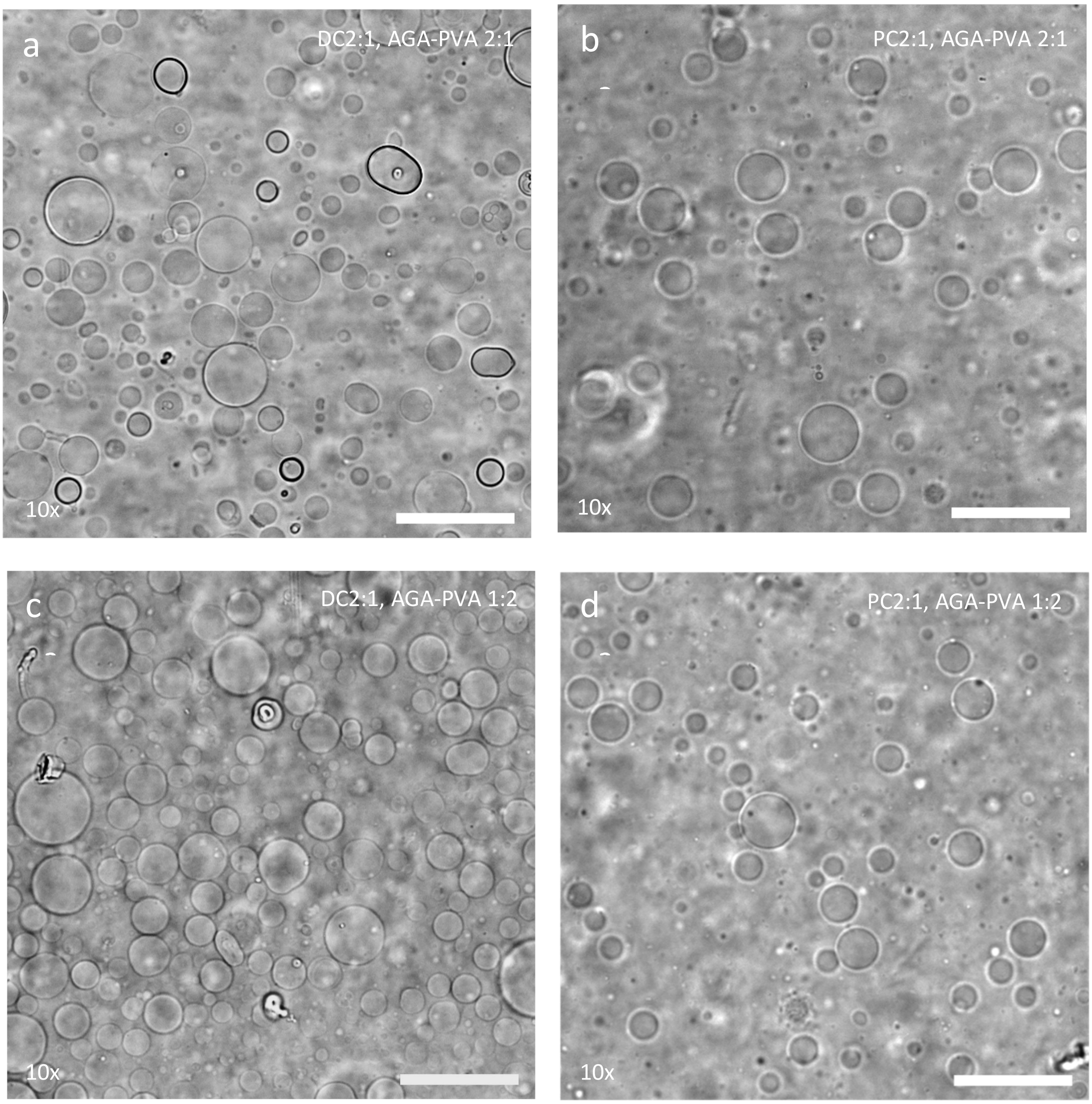

Figure S7. Phase contrast micrographs of GUVs made from DC2:1 on (a) AGA-PVA 2:1, and (c) AGA-PVA 1:2, as well as PC2:1 on (b) AGA:PVA 2:1 and (d) AGA-PVA 1:2. Factors (x) indicate how many times the GUV samples have been concentrated. Scale bar = 50 µm.

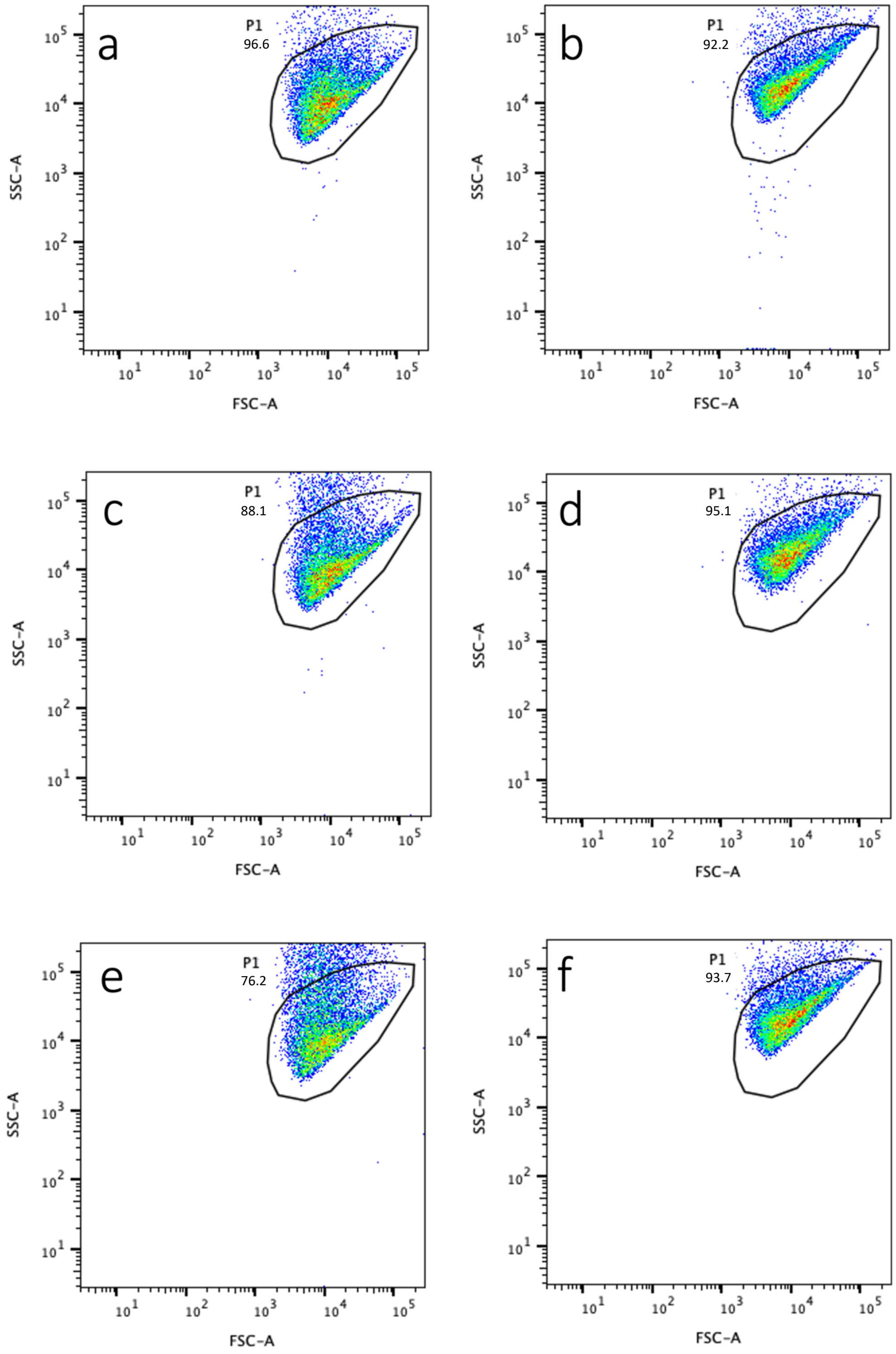

Figure S8. SSC-FSC scatter-plots of GUVs made from DC2:1 on (a) AGA-PVA 2:1, (c) AGA-PVA 1:1, and (e) AGA-PVA 1:2, as well as PC2:1 on (b) AGA:PVA 2:1, (d) AGA-PVA 1:1 and (f) AGA-PVA 1:2.

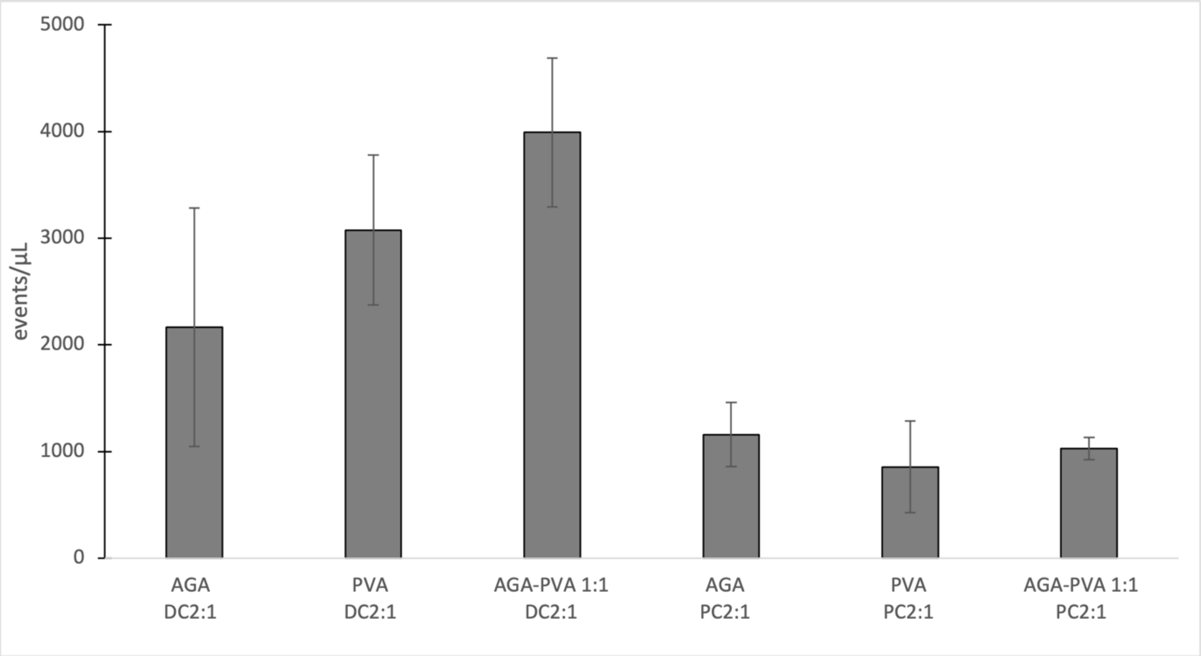

Figure S9. Graph showing yields of DC2:1 GUVs produced using (a) AGA, (b) PVA, and (c) AGA-PVA 1:1, as well as PC2:1 GUVs produced using (d) AGA, (e) PVA, and (f) AGA-PVA 1:1 hydrogels.

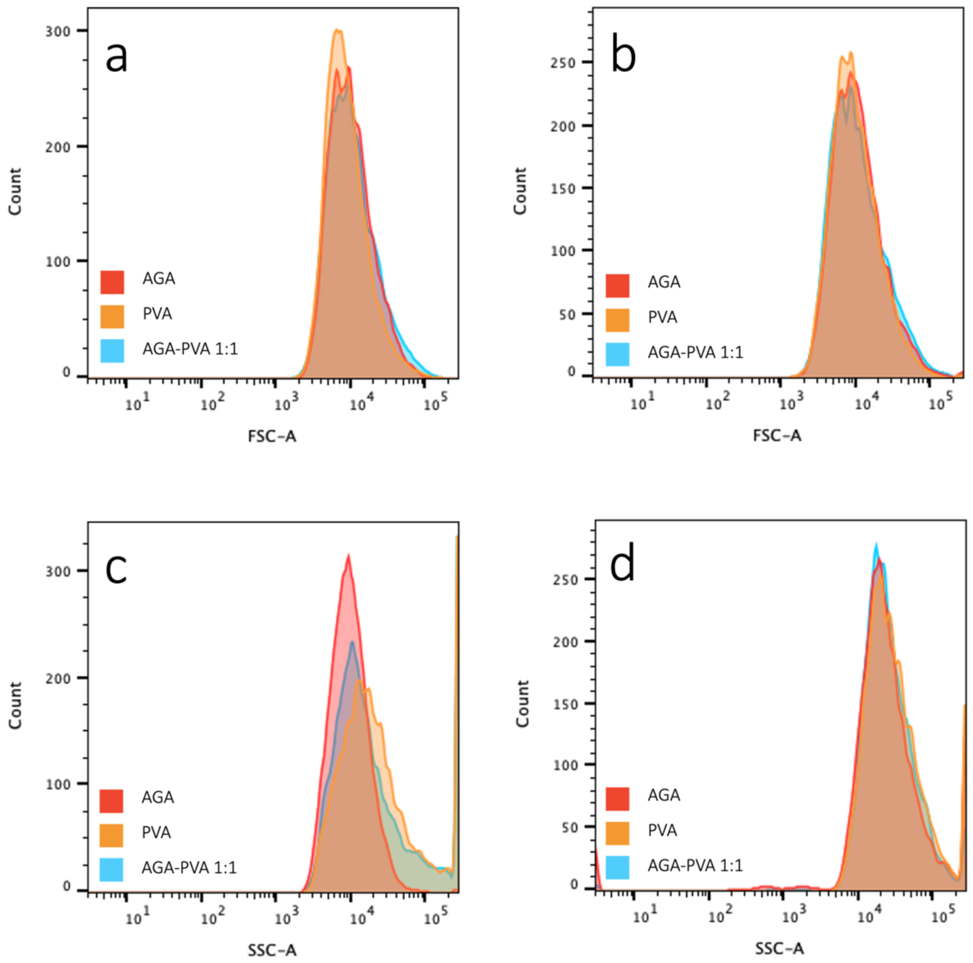

Figure S10. Comparison of size (FSC) distribution of (a) DC2:1 and (b) PC2:1 GUVs and complexity (SSC) distribution of (c) DC2:1 and (d) PC2:1 GUVs produced using AGA, PVA, and AGA-PVA hydrogels. Scatter-plots show the overlay of data from all three types of samples. Green areas indicate where the PVA and AGA-PVA 1:1 profiles overlap.

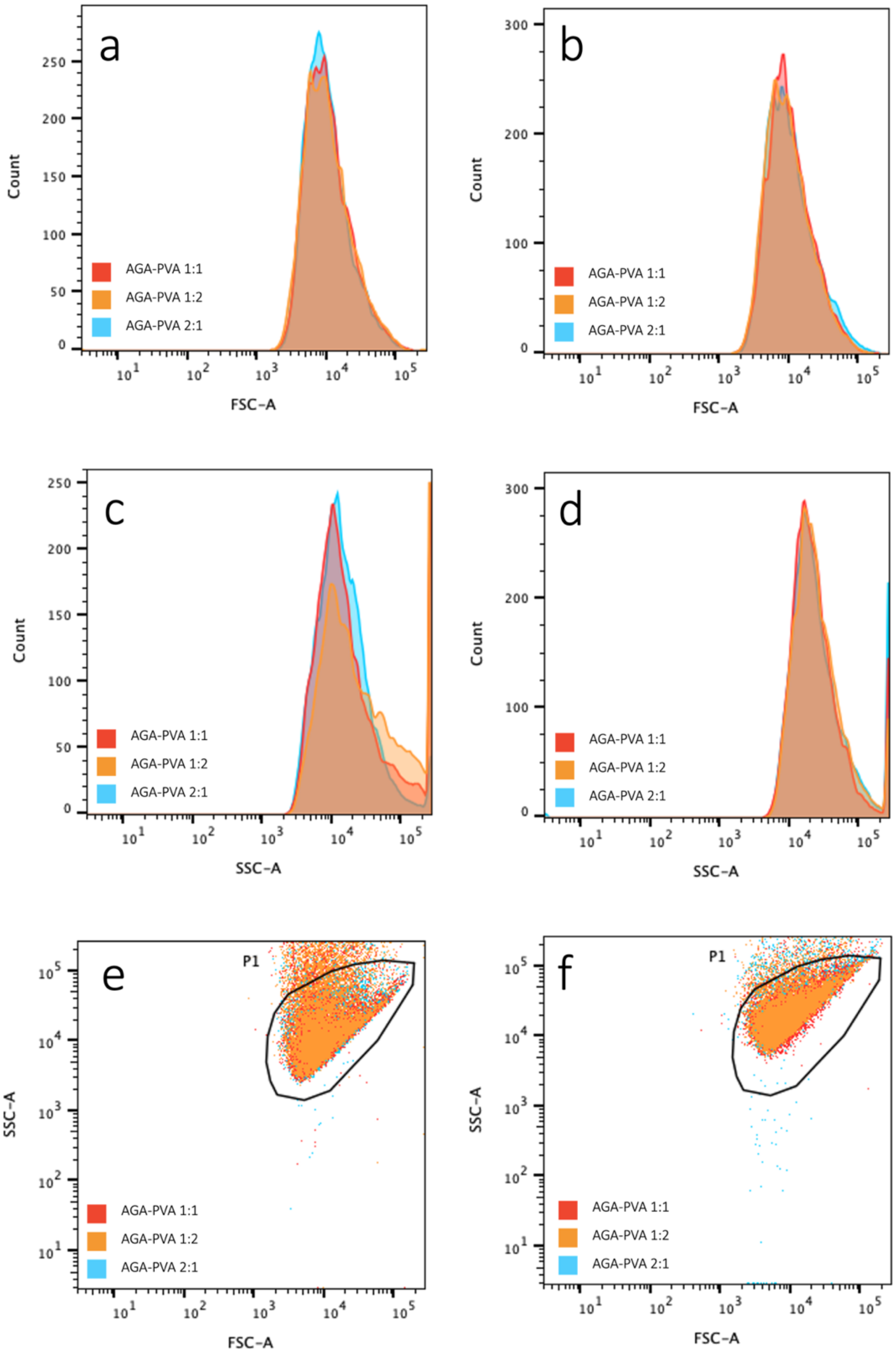

Figure S11. Comparisons of size distribution (FSC) of GUVs made from (a) DC2:1, and (b) PC2:1, as well as complexity distribution (SSC) of GUVs made from (c) DC2:1, and (d) PC2:1. Last row shows SSC-FSC scatter-plots of GUVs made from (e) DC2:1 and (f) PC2:1.

DC2:1 and PC2:1 GUVs produced in polystyrene multi-well plates using AGA, PVA, and AGA-PVA 1:1 hydrogels

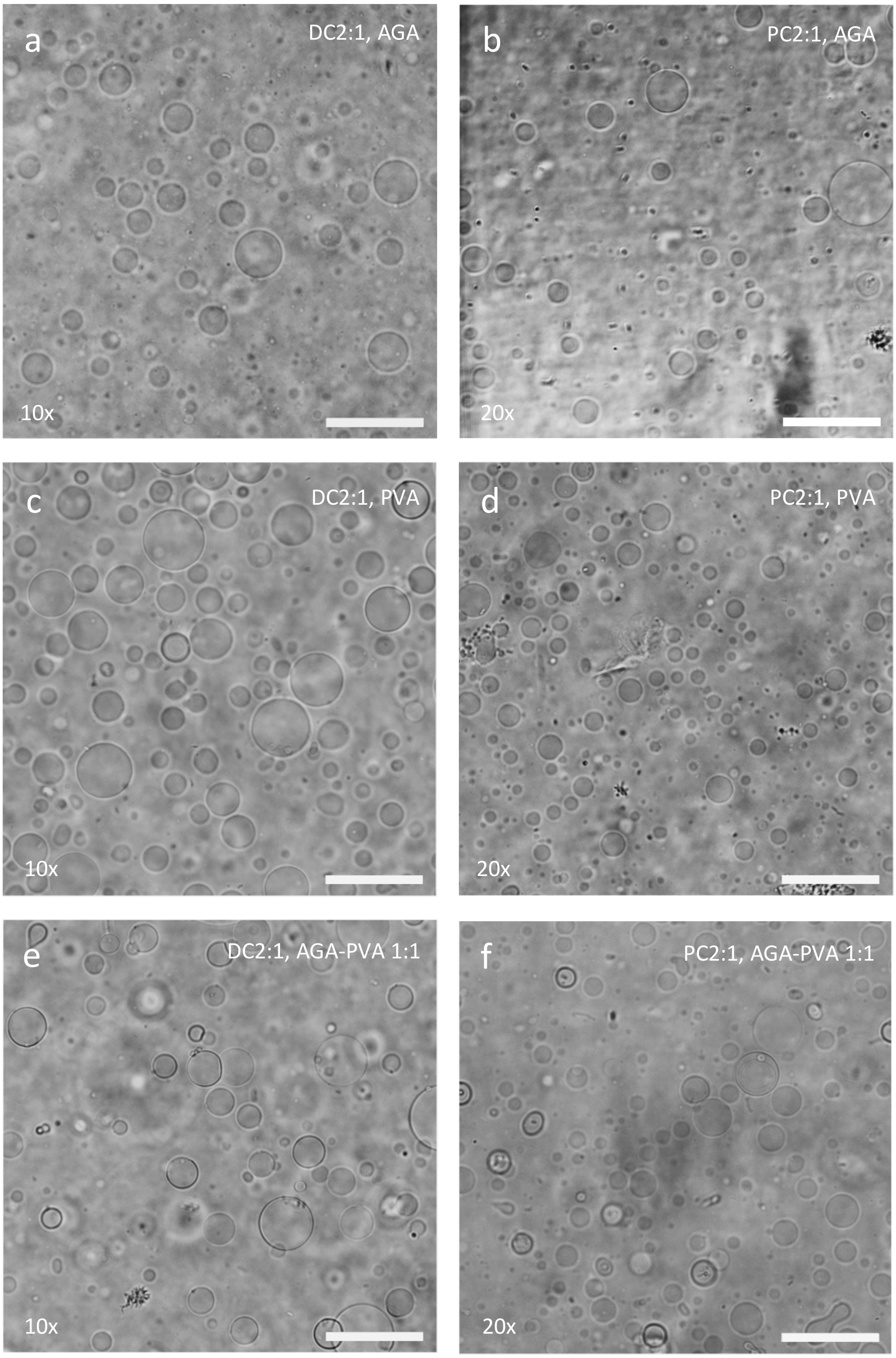

Figure S12. Phase contrast micrographs showing DC2:1 and PC2:1 GUVs produced in polystyrene multi-well plates using various hydrogels. Factors (x) indicate how many times the GUV samples have been concentrated. Scale bar = 50 µm.

DC2:1 GUVs produced on PEG-DA hydrogels

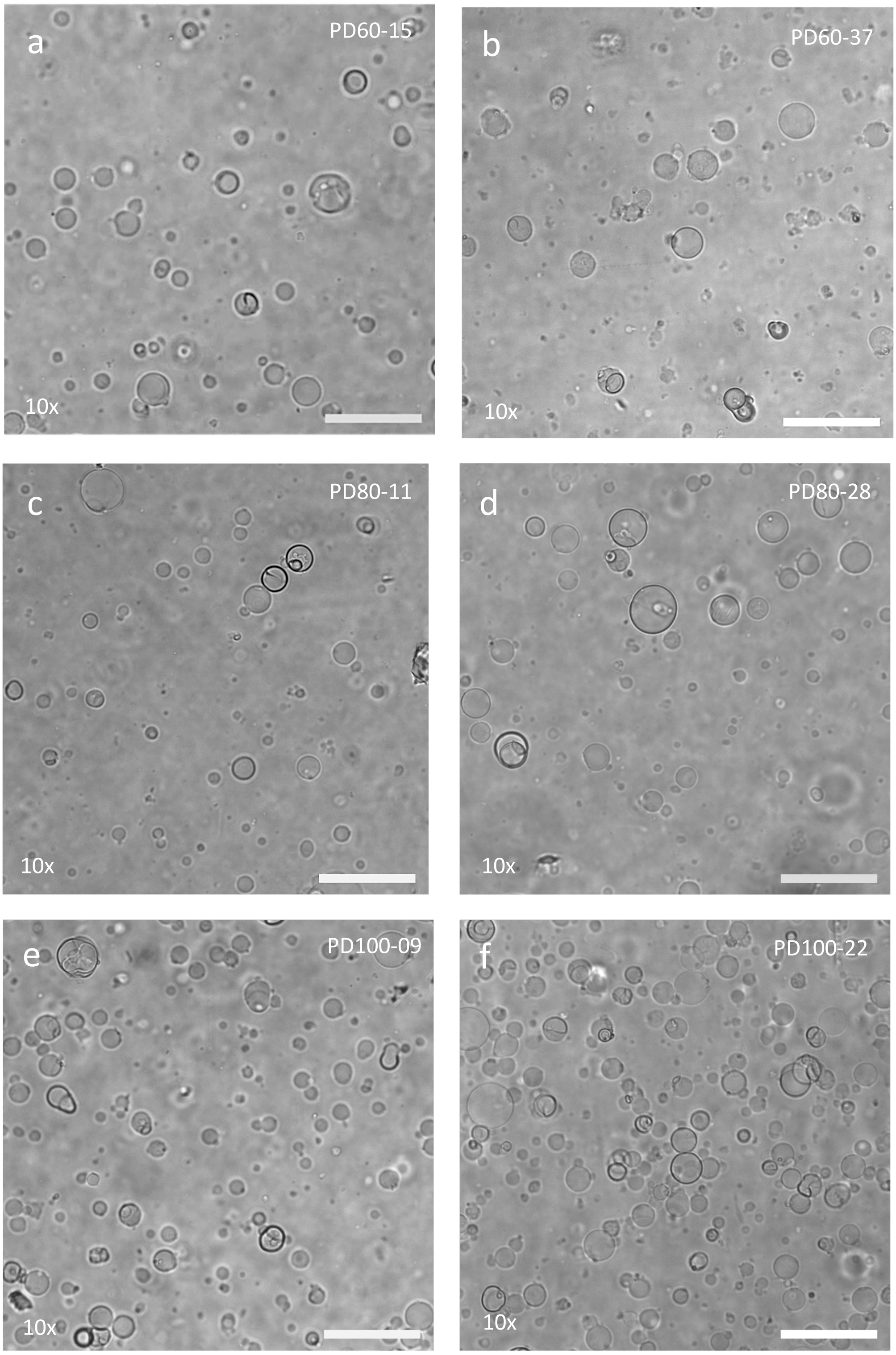

Figure S13. Phase contrast micrographs showing DC2:1 GUVs produced on various PEG-DA hydrogel compositions. Factors (x) indicate how many times the GUV samples have been concentrated. Scale bar = 50 µm.

DC2:1 GUVs produced on cross-linked hyaluronic acid hydrogels

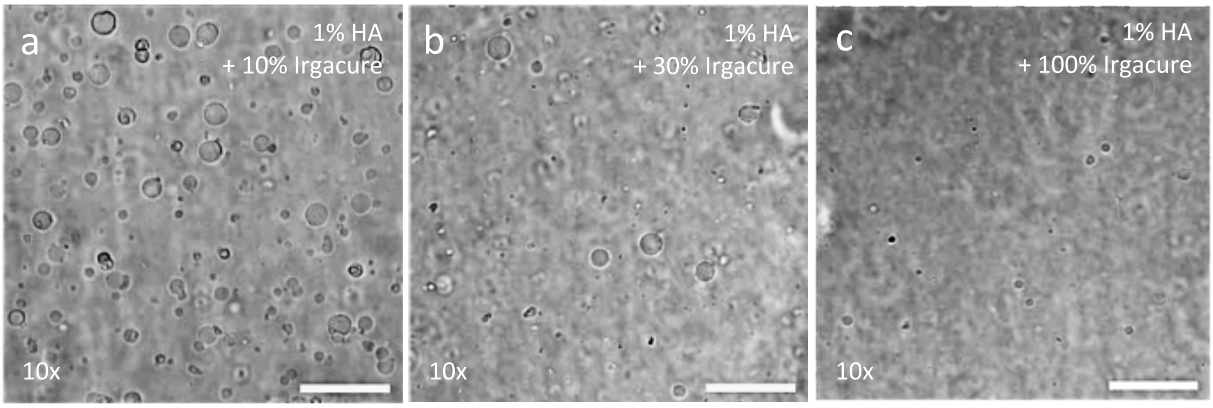

Figure S14. Phase contrast micrographs showing DC2:1 GUVs produced on substrates made from HA and various concentrations of Irgacure. Factors (x) indicate how many times the GUV samples have been concentrated. Scale bar = 50 µm.

DC2:1 Rh-PE GUVs produced on Matrigel hydrogels

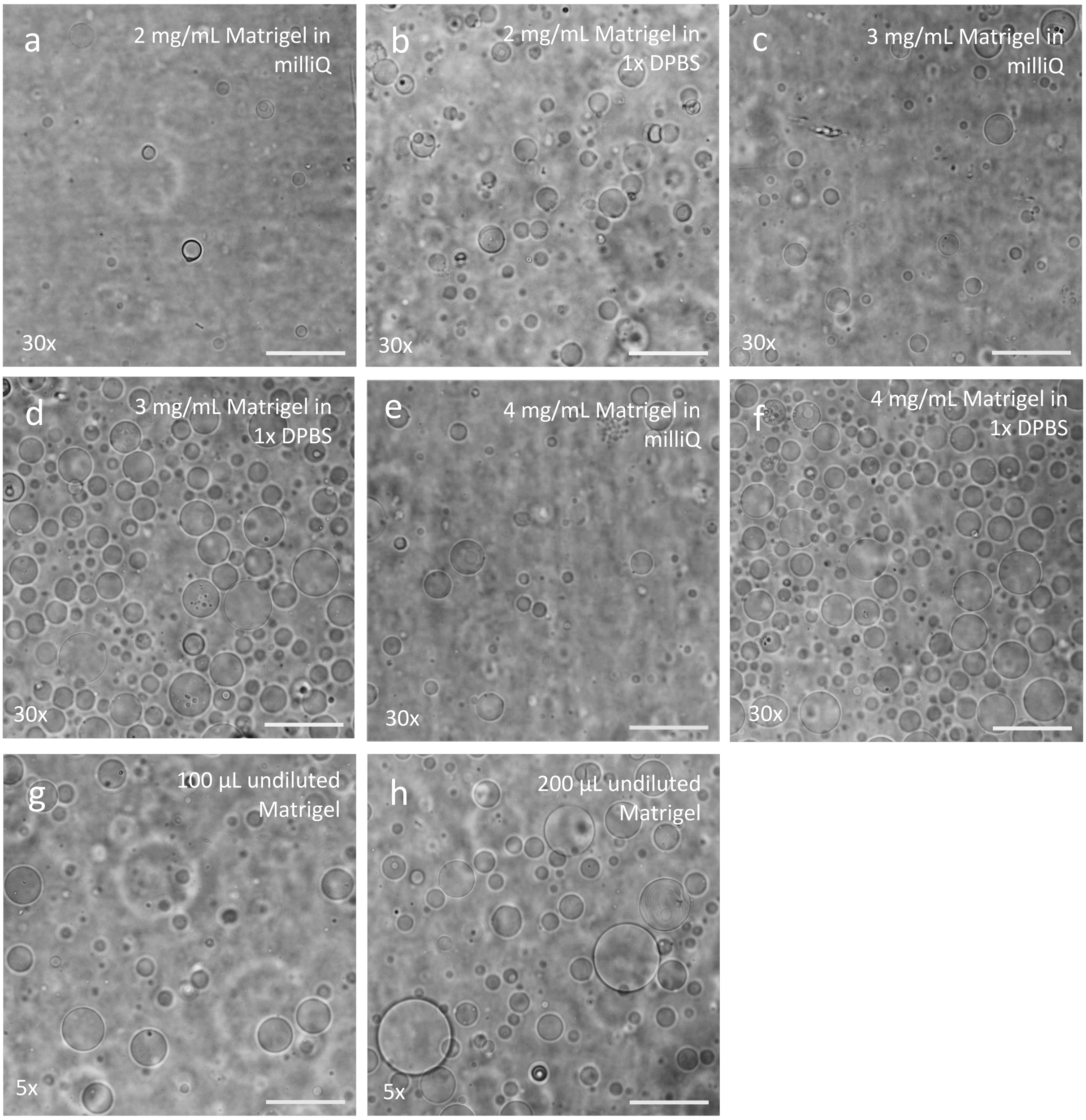

Figure S15. Phase contrast micrographs showing DC2:1 Rh-PE GUVs produced on Matrigel hydrogels at various concentrations. Factors (x) indicate how many times the GUV samples have been concentrated. Scale bar = 50 µm.

DC2:1 GUVs produced on LMW salmon DNA amorphous gels

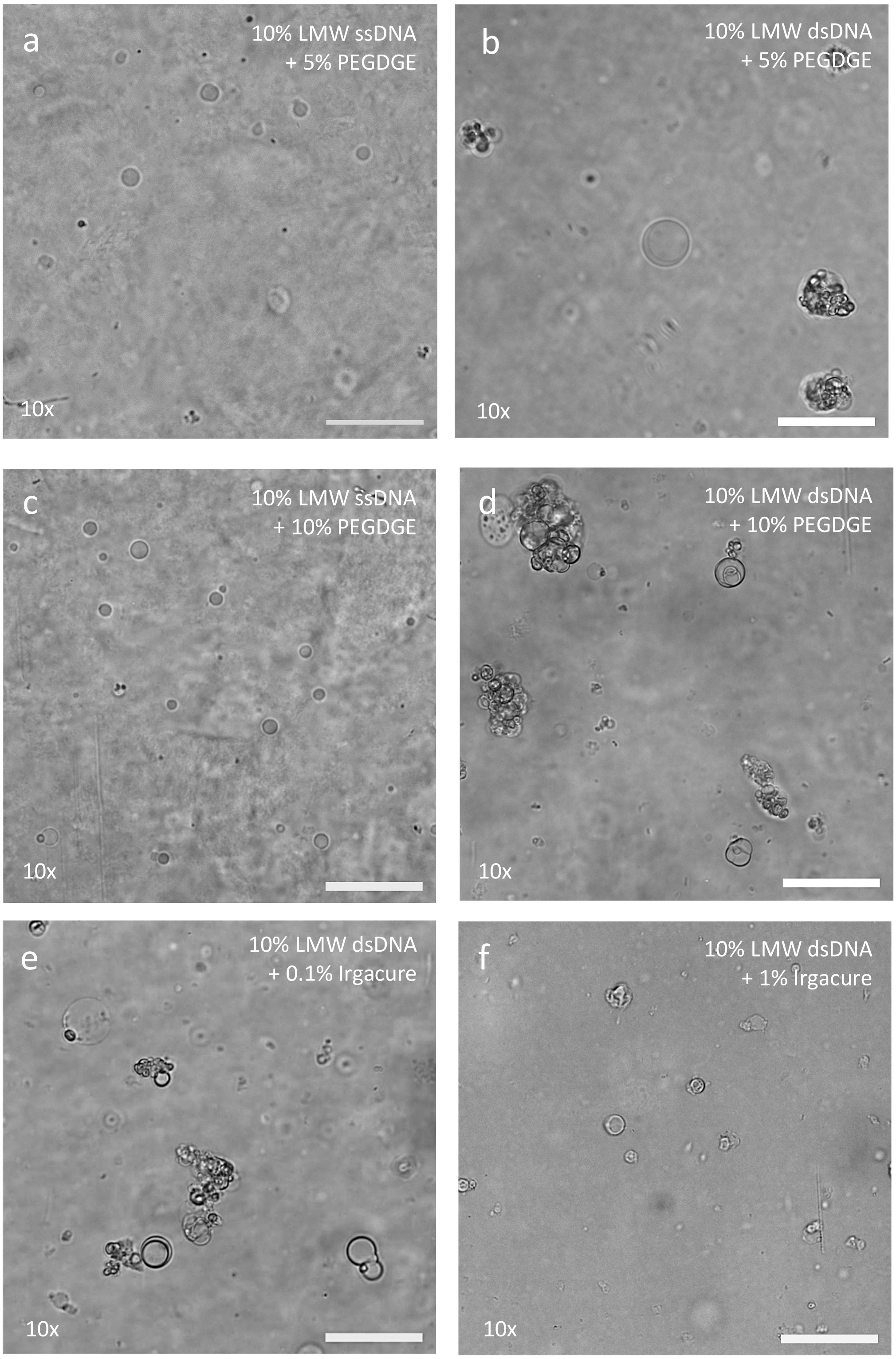

Figure S16. Phase contrast micrographs showing DC2:1 GUVs produced using various LMW DNA substrates. Factors (x) indicate how many times the GUV samples have been concentrated. Scale bar = 50 µm.

DC2:1 GUVs produced on HMW salmon DNA amorphous gels

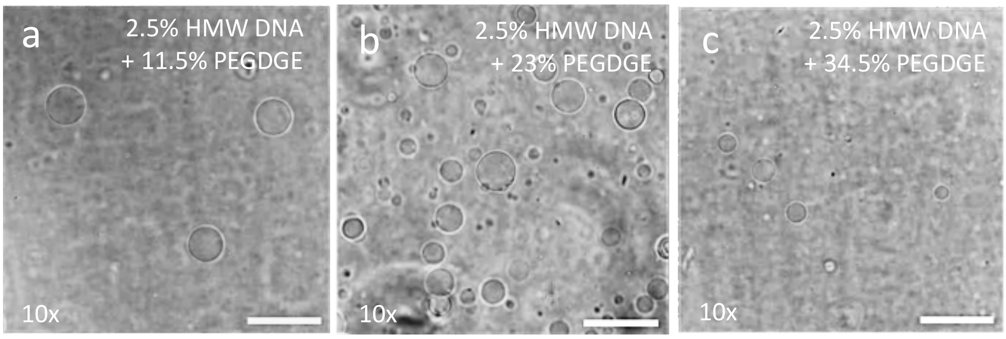

Figure S17. Phase contrast micrographs showing DC2:1 GUVs produced using various HMW DNA substrates. Factors (x) indicate how many times the GUV samples have been concentrated. Scale bar = 50 µm.

Penetration of DC2:1 + Rh-PE into LMW-DNA amorphous gels and HMW-DNA hydrogels

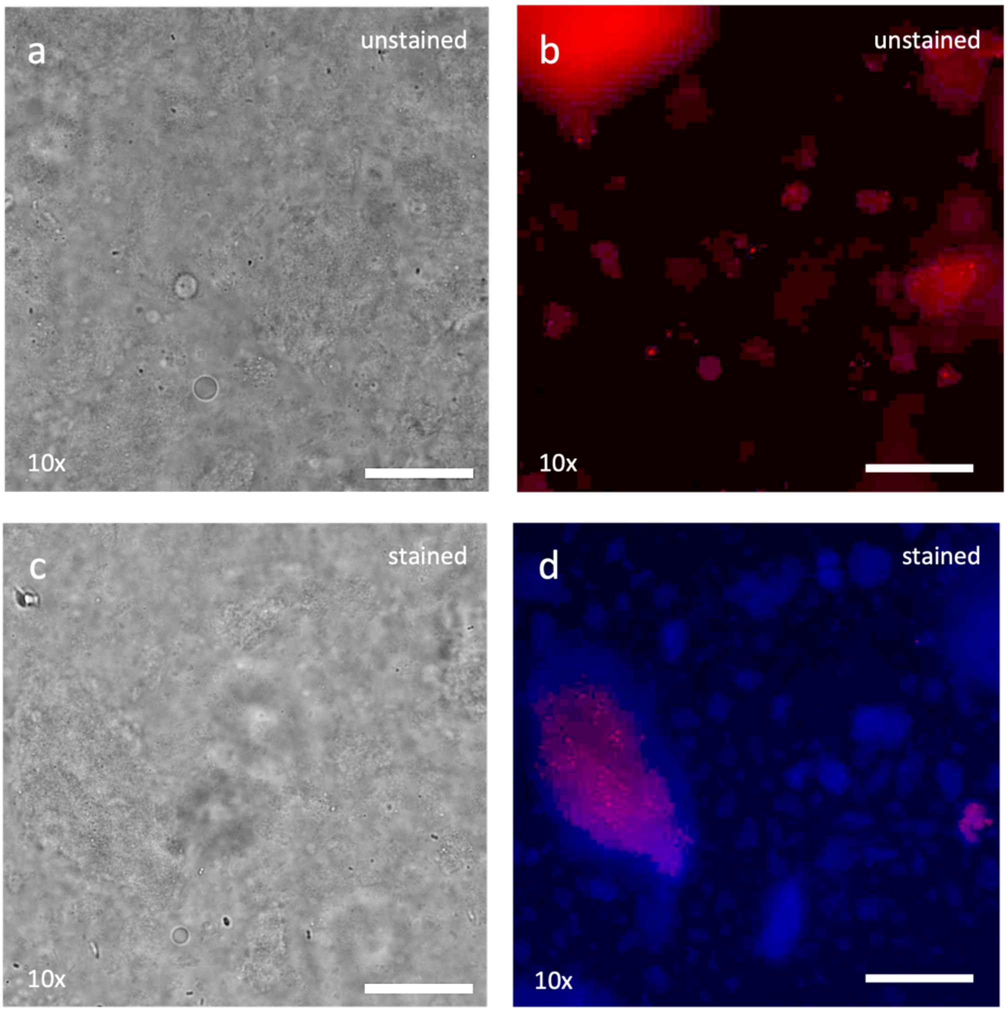

Figure S18. Micrographs showing DC2:1 + Rh-PE GUVs made from 10% LMW-dsDNA + 10% PEGDGE. Phase contrast micrographs of (a) unstained GUVs, and (c) GUVs stained with Hoechst stain as well as corresponding epi-fluorescence micrographs of (b) unstained GUVs, and (d) GUVs stained with Hoechst stain are shown. Factors (x) indicate how many times the GUV samples have been concentrated. Scale bar = 50 µm.

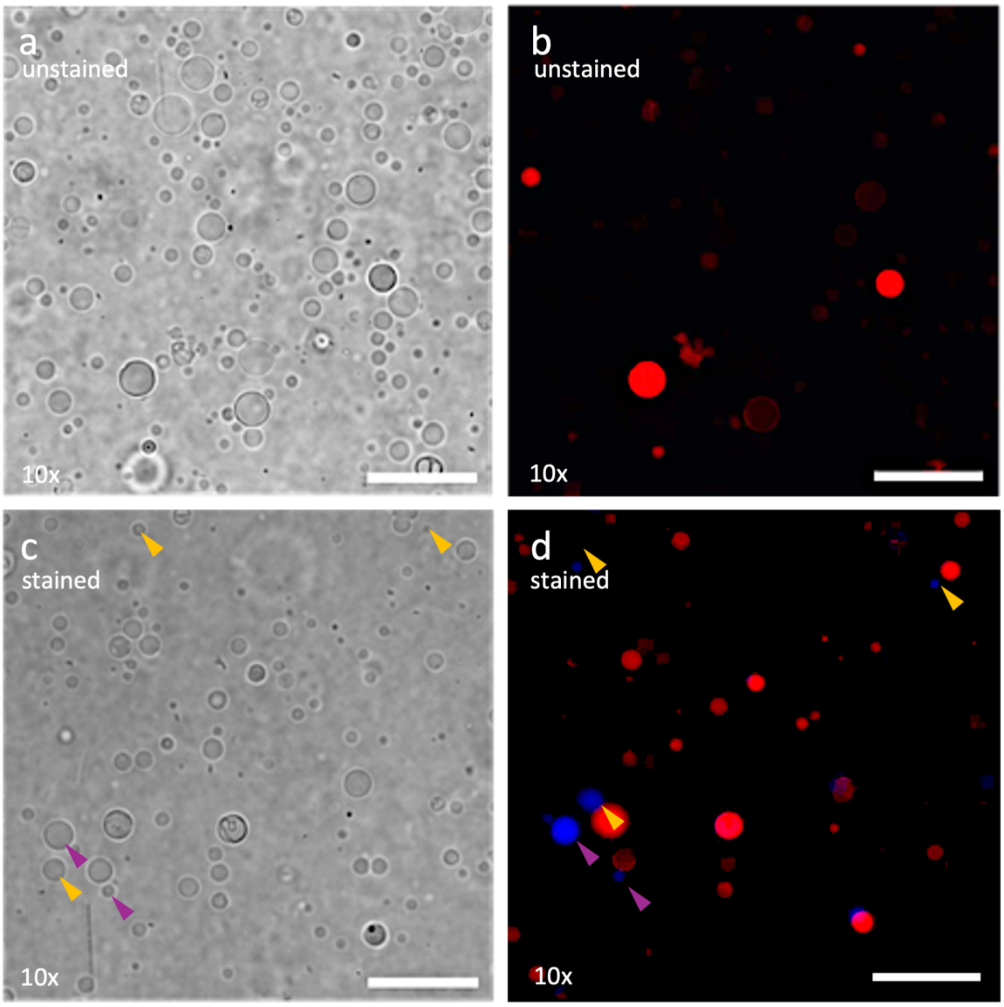

Figure S19. Micrographs showing DC2:1 + Rh-PE GUVs produced on 2.5% HMW-DNA + 23% PEGDGE hydrogels. Phase contrast micrographs of (a) unstained GUVs, and (c) GUVs stained with Hoechst stain as well as corresponding epi-fluorescence micrographs of (b) unstained GUVs, and (d) GUVs stained with Hoechst stain (arrows) are shown. Arrowheads indicate GUVs encapsulating DNA material. Yellow arrows indicate GUVs that have moved. Slight differences in vesicle location between phase contrast and epi-fluorescence images is due to movement of the GUVs. Factors (x) indicate how many times the GUV samples have been concentrated. Scale bar = 50 µm.

GUV yields after each successive use of AGA, PVA, and AGA-PVA hydrogels

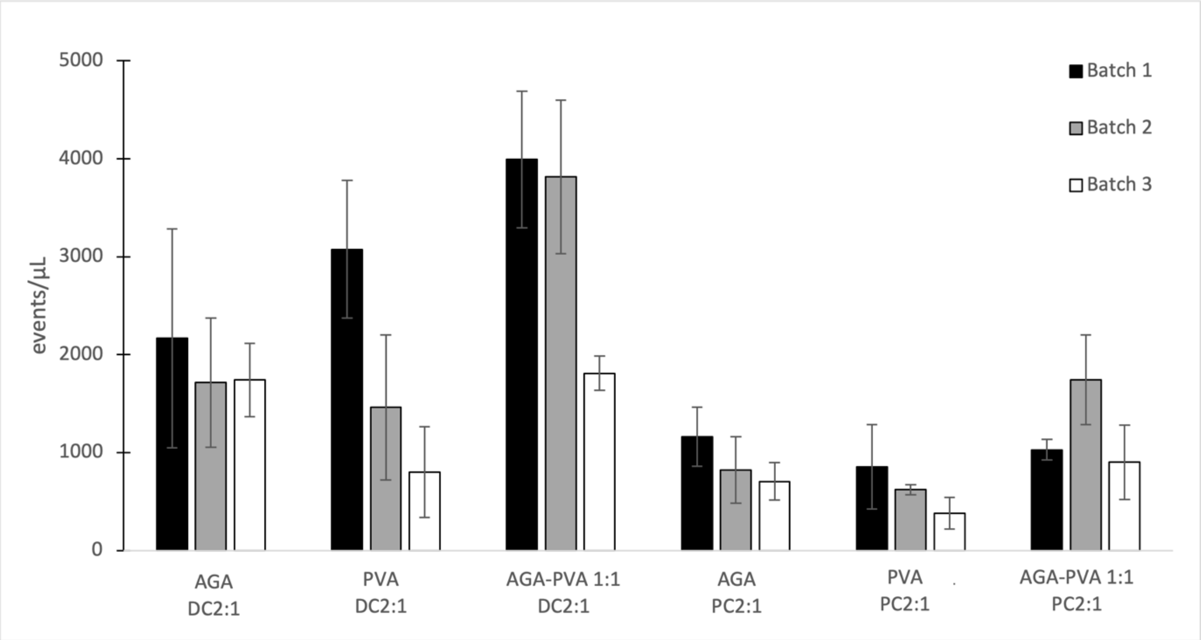

Figure S20. Graph showing GUV yields before and after reusing the AGA, PVA, and AGA-PVA 1:1 hydrogels. Each batch refers to an instance of GUV production.

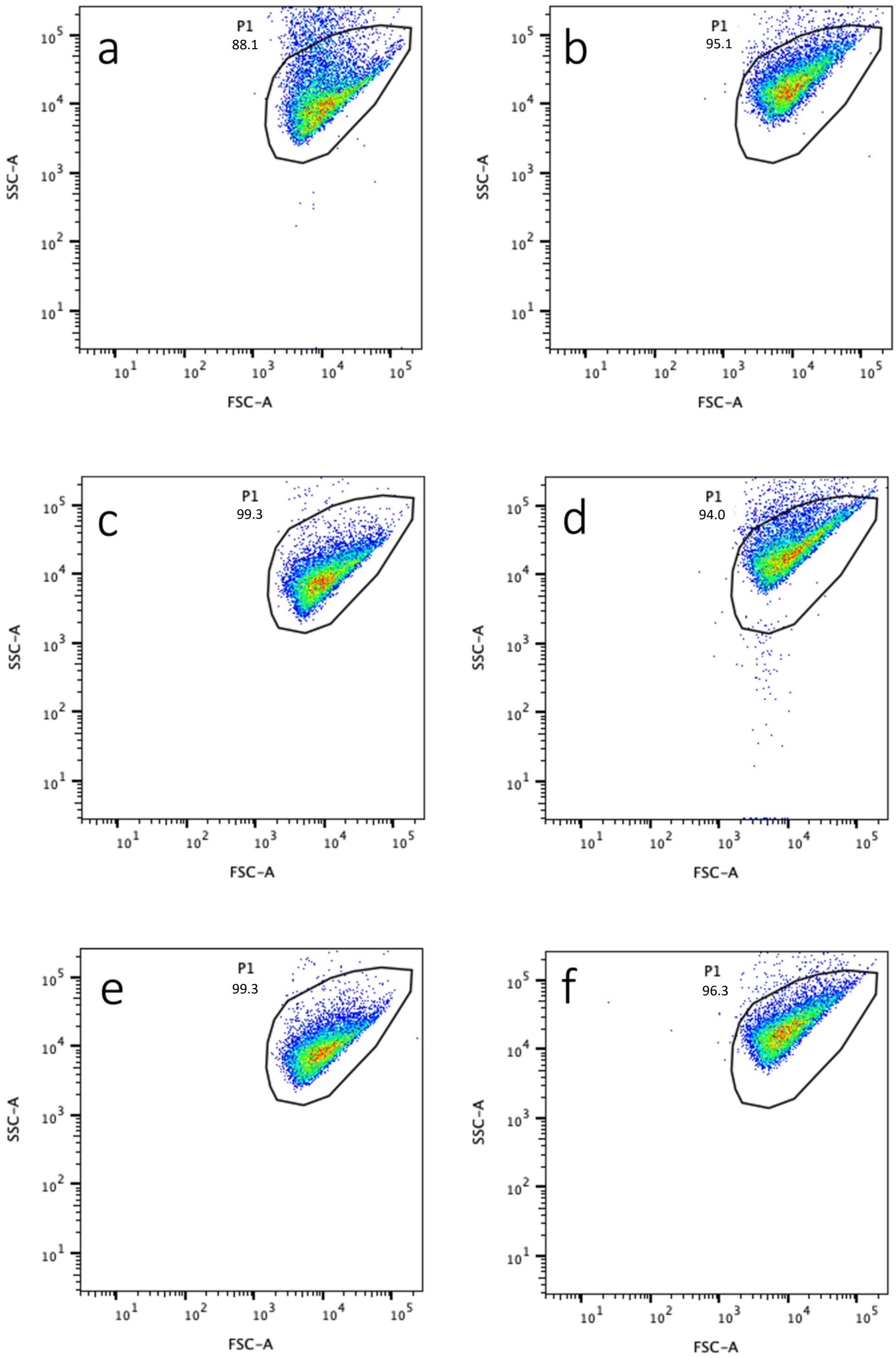

Figure S21. SSC-FSC scatter-plots showing GUVs produced from DC2:1 on AGA-PVA 1:1 (a) used for the first time, (c) reused once, and (e) reused twice, as well as GUVs produced from PC2:1 on AGA-PVA 1:1 (b) used for the first time, (d) reused once, and (f) reused twice.

Determination of salmon DNA fragment size

To ascertain the size of the salmon DNA fragments used, both high and low molecular weight salmon DNA was analysed by gel electrophoresis in 1% agarose-TAE. To confirm the electrophoretic data, a Genomic DNA Screen Tape Assay was also performed using a TapeStation 4150 device (Agilent). Both high and low molecular weight salmon DNA used for this analysis were dissolved in nuclease-free water to a final concentration of 0.2 mg/mL. *TapeStation Controller Software* (version 5.1) and *TapeStation Analysis Software* (version 5.1) were used to control the device and process the data. TapeStation genomic DNA ScreenTape (Part. No. 5067-5365) as well as buffer and ladder (Part. No. 5067-5366) were purchased from Agilent Technologies, USA.

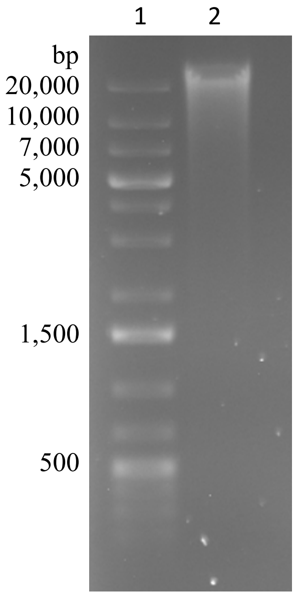

1. GeneRuler 1kb Plus DNA ladder
2. Sigma-Aldrich HMW Salmon DNA (D1626)

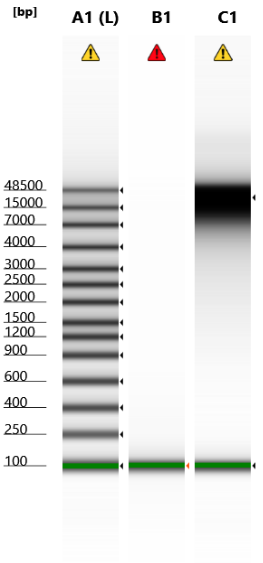

Compact Peak Table

Well	Sample Description	Size [bp]	Calibrated Conc. [ng/µl]	Assigned Conc. [ng/µl]	%Integrated Area	From [bp]	To [bp]	Peak Comment	Observations
A1	Ladder	100	8.50	8.50	-	67	157		Lower Marker
A1	Ladder	250	5.68	-	6.87	183	318		
A1	Ladder	400	6.42	-	7.77	335	504		
A1	Ladder	600	6.68	-	8.09	513	762		
A1	Ladder	900	6.40	-	7.74	769	1044		
A1	Ladder	1200	6.61	-	8.00	1044	1342		
A1	Ladder	1500	7.22	-	8.73	1342	1792		
A1	Ladder	2000	6.52	-	7.89	1792	2253		
A1	Ladder	2500	6.46	-	7.82	2253	2758		
A1	Ladder	3000	6.83	-	8.27	2758	3560		
A1	Ladder	4000	6.42	-	7.76	3560	5495		
A1	Ladder	7000	6.38	-	7.72	5495	10943		
A1	Ladder	15000	5.65	-	6.84	10943	22849		
A1	Ladder	48500	5.21	-	6.31	22849	78534		
A1	Ladder	-	-	-	-	-	-		Sample Well
B1	LMW DNA	100	8.50	8.50	-	65	157		Lower Marker
C1	HMW DNA	100	8.50	8.50	-	65	152		Lower Marker
C1	HMW DNA	23342	69.9	-	93.47	5690	>60000		

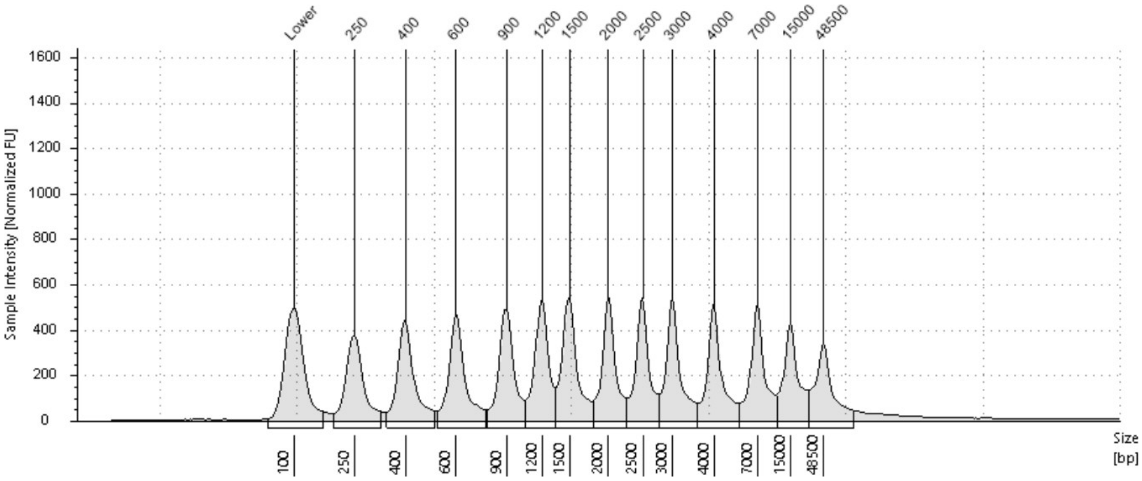

Peak Table

Size [bp]	Calibrated Conc. [ng/µl]	Assigned Conc. [ng/µl]	% Integrated Area	From [bp]	To [bp]	Peak Comment	Observations
100	8.50	8.50	-	67	157		Lower Marker
250	5.68	-	6.87	183	318		
400	6.42	-	7.77	335	504		
600	6.68	-	8.09	513	762		
900	6.40	-	7.74	769	1044		
1200	6.61	-	8.00	1044	1342		
1500	7.22	-	8.73	1342	1792		
2000	6.52	-	7.89	1792	2253		
2500	6.46	-	7.82	2253	2758		
3000	6.83	-	8.27	2758	3560		
4000	6.42	-	7.76	3560	5495		
7000	6.38	-	7.72	5495	10943		
15000	5.65	-	6.84	10943	22849		
48500	5.21	-	6.31	22849	78534		
-	-	-	-	-	-		Sample Well

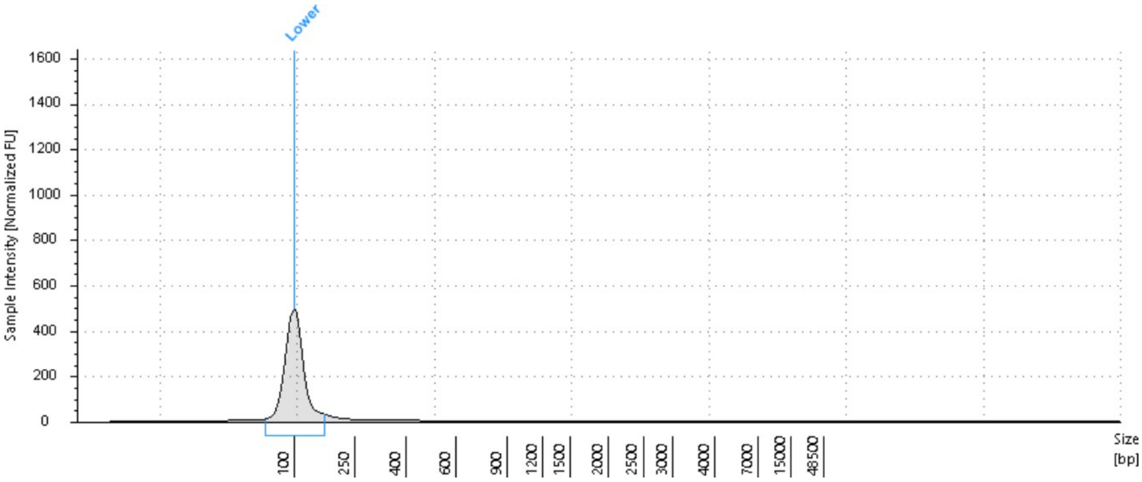

Peak Table

Size [bp]	Calibrated Conc. [ng/µl]	Assigned Conc. [ng/µl]	% Integrated Area	From [bp]	To [bp]	Peak Comment	Observations
100	8.50	8.50	-	65	157		Lower Marker

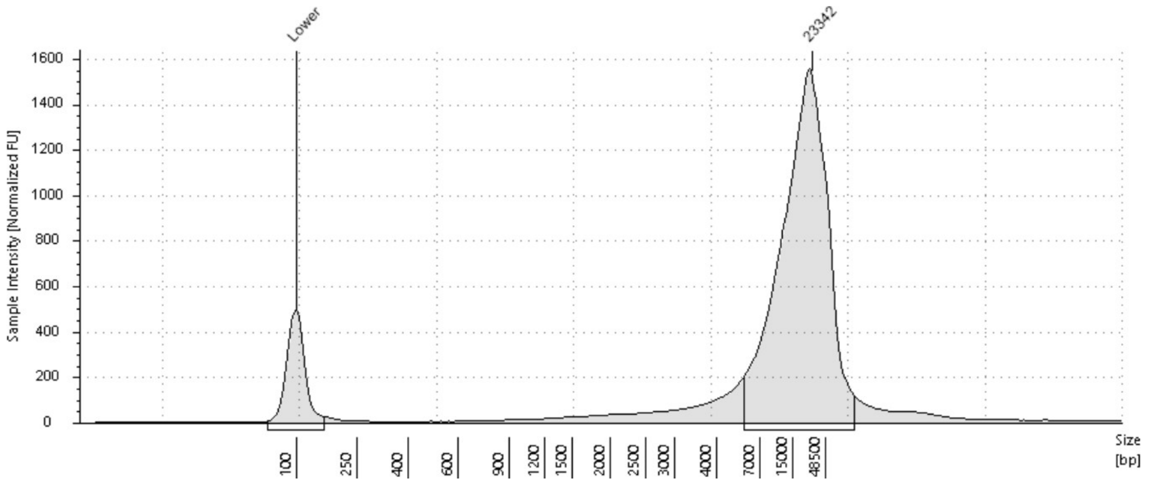

Peak Table

Size [bp]	Calibrated Conc. [ng/µl]	Assigned Conc. [ng/µl]	% Integrated Area	From [bp]	To [bp]	Peak Comment	Observations
100	8.50	8.50	-	65	152		Lower Marker
23342	69.9	-	93.47	5690	>60000		
-	-	-	-	-	-		Sample Well

Figure S22. Genomic DNA Screen Tape Assay evaluation of DNA fragment lengths in Low molecular weight DNA (LMW-DNA) and high molecular weight DNA (HMW-DNA) from salmon.

Table and Figure Titles

Table S1: Summary of selected reports describing hydrogel-assisted GUV production.

Table S2: Summary of materials and their crosslinking agents used for producing substrates in our work.

Table S3: Summary of lipids and block co-polymers used for producing GUVs in our work.

Figure S1. Render showing design of poly(lactic acid) mould for casting PDMS mould.

Figure S2. SSC-FSC scatter-plots of GUVs made from (a) DC1:0, (b) PC1:0, (c) DC2:1, (d) PC2:1, (e) DP1:0, and (f) PPC1:0 on AGA hydrogels.

Figure S3. Phase contrast micrographs of GUVs produced from various membrane materials on PVA hydrogels.

Figure S4. SSC-FSC scatter-plots of GUVs produced from (a) DC1:0, (b) PC1:0, (c) DC2:1, (d) PC2:1, (e) DP1:0, and (f) PPC1:0 on PVA hydrogels.

Figure S5. Comparisons of (a) size distribution (FSC), (b) complexity distribution, and (c) SSC-FSC scatter-plots of GUVs made on PVA

Figure S6. Phase contrast micrographs showing DC2:1 and PC2:1 GUVs produced various hydrogels.

Figure S7. Phase contrast micrographs of GUVs made from DC2:1 on (a) AGA-PVA 2:1, and (c) AGA-PVA 1:2, as well as PC2:1 on (b) AGA:PVA 2:1 and (d) AGA-PVA 1:2.

Figure S8. SSC-FSC scatter-plots of GUVs made from DC2:1 on (a) AGA-PVA 2:1, (c) AGA-PVA 1:1, and (e) AGA-PVA 1:2, as well as PC2:1 on (b) AGA:PVA 2:1, (d) AGA-PVA 1:1 and (f) AGA-PVA 1:2.

Figure S9. Graph showing yields of DC2:1 GUVs produced using (a) AGA, (b) PVA, and (c) AGA-PVA 1:1, as well as PC2:1 GUVs produced using (d) AGA, (e) PVA, and (f) AGA-PVA 1:1 hydrogels.

Figure S10. Comparison of size (FSC) distribution of (a) DC2:1 and (b) PC2:1 GUVs and complexity (SSC) distribution of (c) DC2:1 and (d) PC2:1 GUVs produced using AGA, PVA, and AGA-PVA hydrogels.

Figure S11. Comparisons of size distribution (FSC) of GUVs made from (a) DC2:1, and (b) PC2:1, as well as complexity distribution (SSC) of GUVs made from (c) DC2:1, and (d) PC2:1.

Figure S12. Phase contrast micrographs showing DC2:1 and PC2:1 GUVs produced in polystyrene multi-well plates using various hydrogels.

Figure S13. Phase contrast micrographs showing DC2:1 GUVs produced on various PEG-DA hydrogel compositions.

Figure S14. Phase contrast micrographs showing DC2:1 GUVs produced on substrates made from HA and various concentrations of Irgacure.

Figure S15. Phase contrast micrographs showing DC2:1 Rh-PE GUVs produced on Matrigel hydrogels at various concentrations.

Figure S16. Phase contrast micrographs showing DC2:1 GUVs produced using various LMW DNA substrates.

Figure S17. Phase contrast micrographs showing DC2:1 GUVs produced using various HMW DNA substrates.

Figure S18. Micrographs showing DC2:1 + Rh-PE GUVs made from 10% LMW-dsDNA + 10% PEGDGE.

Figure S19. Micrographs showing DC2:1 + Rh-PE GUVs produced on 2.5% HMW-DNA + 23% PEGDGE hydrogels.

Figure S20. Graph showing GUV yields before and after reusing the AGA, PVA, and AGA-PVA 1:1 hydrogels.

Figure S21. SSC-FSC scatter-plots showing GUVs produced from DC2:1 on AGA-PVA 1:1 (a) used for the first time, (c) reused once, and (e) reused twice, as well as GUVs produced from PC2:1 on AGA-PVA 1:1 (b) used for the first time, (d) reused once, and (f) reused twice.

Figure S22. Genomic DNA Screen Tape Assay evaluation of DNA fragment lengths in Low molecular weight DNA (LMW-DNA) and high molecular weight DNA (HMW-DNA) from salmon.
